# An update to the taxonomy and distribution of the Arabian *Tapinoma* Foerster, 1850 (Hymenoptera: Formicidae) with an illustrated key and remarks on habitats

**DOI:** 10.3897/BDJ.9.e66058

**Published:** 2021-05-27

**Authors:** Mahmoud M Abdel-Dayem, Hathal Mohammed Al Dhafer, Abdulrahman S Aldawood, Mostafa R Sharaf

**Affiliations:** 1 Plant Protection Department, College of Food and Agricultural Sciences, King Saud University, Riyadh, Saudi Arabia Plant Protection Department, College of Food and Agricultural Sciences, King Saud University Riyadh Saudi Arabia; 2 Entomology Department, Faculty of Science, Cairo University, Giza, Egypt Entomology Department, Faculty of Science, Cairo University Giza Egypt; 3 King Saud University, College of Food and Agriculture Sciences, Riyadh, Saudi Arabia King Saud University, College of Food and Agriculture Sciences Riyadh Saudi Arabia

**Keywords:** Afrotropical Region, Dolichoderinae, endemic, Middle East

## Abstract

**Background:**

*Tapinoma* Foerster belongs to the ant subfamily Dolichoderinae and the vast majority of its species are arboreal or generalised foragers. The genus is composed of 70 described species, 22 known subspecies and six valid fossil species worldwide, while from the Arabian Peninsula, three species have been recorded so far.

**New information:**

Ants of the genus *Tapinoma* of the Arabian Peninsula are reviewed, keyed and illustrated, based on the worker caste. Three species are diagnosed, *T. melanocephalum* (Fabricius, 1793), *T. simrothi* Krausse, 1911 and *T. wilsoni* Sharaf & Aldawood, 2012. We present the first illustrated key to the Arabian *Tapinoma*, enhanced by automontage images to facilitate species recognition. New distributional data for species are presented, based on recently-collected material from the region and literature records. Information on habitats' preference and biology of species are given.

## Introduction

**Figure 1.**
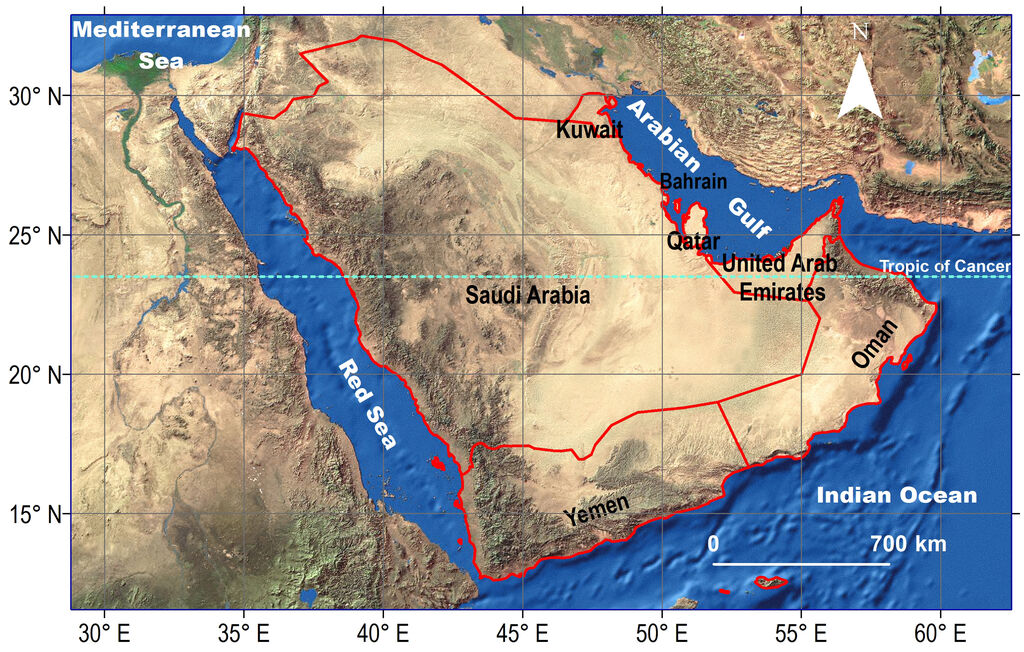
Map of the Arabian Peninsula.

The Arabian Peninsula, in western Asia, covers a surface area of 3.2 million km^2^ comprising Bahrain, Kuwait, Oman, Qatar, Saudi Arabia, United Arab Emirates (UAE) and Yemen (Fig. 1). It turns out to be a very interesting biogeographic area, since it straddles the Afrotropical, the Palaearctic and, to a lesser extent, the Oriental realms (Larsen 1984, Hölzel 1998). With its location at the interchange of three biogeographic realms, the Arabian Peninsula shares faunal elements from those three Regions (Zunino and Zullini 1995, Abdel-Dayem et al. 2018, Abdel-Dayem et al. 2019, Ziani et al. 2019, Abdel-Dayem et al. 2020, Letardi et al. 2020). Several faunistic contributions have shed light on zoogeography of the Arabian ants, indicating the predominance of Afrotropical species in the south-western mountains of the Arabian Peninsula and extending southwards to Yemen and eastwards to the Dhofar Province of Oman. While the species from the Palearctic prevail outside this mountainous range including the vast desert regions of the central and northern Arabia, in addition to the countries of the eastern region (Kuwait, Bahrain, Oman, Qatar and UAE) (Collingwood 1985, Collingwood and Agosti 1996, Collingwood et al. 1997, Collingwood et al. 2011, Sharaf et al. 2014, Sharaf et al. 2018, Sharaf et al. 2020a, Sharaf et al. 2020b, Sharaf et al. 2021).

The genus *Tapinoma* was described with the type-species *T. collina* (junior synonym of *Tapinoma erraticum*), by monotypy (Foerster 1850). With 71 described species, 24 known subspecies and six valid fossil species, *Tapinoma* is one of the largest genera of the subfamily Dolichoderinae (Bolton 2021). The vast majority of species are arboreal (Shattuck 1992) or generalised foragers (Brown 2000), with a remarkable preference for attending honeydew-producing insects (Venkataramaiah and Rehman 1989).

The genus was recorded and keyed for the first time from the Arabian Peninsula by two species, *Tapinoma melanocephalum* (Fabricius 1793) and *T. simrothi* Krausse 1911, collected from the Kingdom of Saudi Arabia (KSA) and Oman (Collingwood 1985). The faunal study of the ant species of the Arabian Peninsula (Collingwood and Agosti 1996) reported *T. melanocephalum* from Yemen and *T. simrothi* from Kuwait and Yemen. *Tapinoma melanocephalum* and *T. simrothi* were collected from UAE by Collingwood et al. (1997), Collingwood et al. (2011). The rare species *T. wilsoni* Sharaf & Aldawood, 2012 was described from the Al Sarawat Mountains of Saudi Arabia, based on the worker caste (Sharaf et al. 2012). Recently, Al-keridis et al. (2021) described the queen of *T. wilsoni* and presented the first key of the Arabian *Tapinoma*, based on the queen caste. More recently, Sharaf et al. (2020b) reported *T. melanocephalum* and *T. simrothi* from the State of Qatar.

*Tapinoma melanocephalum* and *T. simrothi*, are widely spread either in urban sites or wild habitats of the Arabian Peninsula, including agricultural fields and date palm farms (Collingwood 1985). The two species are frequently collected by research projects concerned with the environment and agriculture of the Region (Sharaf et al. 2012). Due to the documented relationships between numerous dolichoderine ants (e.g. *T. simrothi*) and a wide range of sap-sucking insects, such as mealybugs (e.g. Sharaf and Aldawood 2011, Xu et al. 2019) and aphids (e.g. Addicott 1978, Venkataramaiah and Rehman 1989), ecological and biological studies of these ant species are necessary from an agricultural perspective, since it is likely that they contribute in the protection and distribution of such agricultural pests.

The aims of this study are to provide an illustrated key to facilitate *Tapinoma* species recognition, study the geographical distribution of species and give notes on species habitat preference.

## Materials and methods

In total, 457 specimens were collected from countries of the Arabian Peninsula (Suppl. material 1) using different collecting techniques including hand picking (HP), Light trap (LT), Malaise trap (MT), pitfall trap (PT), sifting tray (SF), sweeping net (SW) and Yellow Pan trap (YPT). Specimens were examined using a stereomicroscope Leica M205 with an optical resolution of 0.952 µm, 20.5:1 zoom, 7.8x to 160x magnification and up to 1050 lp/mm resolution (with 2.0x objective). Digital colour images of each species were taken using a Leica DFC450 digital camera with a Leica Z16 APO microscope and LAS (v.3.8) software. The images are available online on AntWeb (http://www.AntWeb.org) and are accessible through unique specimen identifiers (e.g. CASENT0906356). The species names follow the online catalogue of ants of the world (Bolton 2021). Distribution maps were made using DIVA-GIS (version 7.5.0.0). Throughout the work, “w” stands for worker/workers, “q” for queen castes.

### Abbreviations of Museums

**BMNH**, The Natural History Museum, London, United Kingdom.

**CASC**, California Academy of Sciences, San Francisco, USA.

**KSMA**, King Saud University Museum of Arthropods, College of Food and Agriculture Sciences, King Saud University, Riyadh, Saudi Arabia.

**JWPC**, James Wetterer Private Collection, Wilkes Honors College, Florida Atlantic University, 5353 Parkside Drive, Jupiter, FL 33458, USA

**WMLC**, World Museum Liverpool, Liverpool, U.K.

## Taxon treatments

### Tapinoma melanocephalum

(Fabricius, 1793)

14F387D2-3EC4-5E28-B1E3-6CAEB4F14596

https://www.gbif.org/species/1316805

Formica melanocephala Fabricius, 1793: 353 (w.) [Note: neotype w. designated by: Guerrero 2018: 499. Colombia: Magdalena. Guerrero, 2018: 499, also nominates “paraneotypes”. Under ICZN rules no such category exists.] French Guiana. Neotropic.
Formica melanocephala Fabricius, 1793: 353 (w.) [Note: neotype w. designated by: Guerrero 2018: 499. Colombia: Magdalena. Guerrero, 2018: 499, also nominates “paraneotypes”. Under ICZN rules no such category exists.] French Guiana. Neotropic.

#### Materials

- **Type status:** Other material. **Occurrence:** recordedBy: Mostafa Sharaf; individualCount: 1; sex: worker; lifeStage: adult; **Taxon:** scientificName: Tapinoma melanocephalum; **Location:** country: Oman; locality: Qurayat; verbatimElevation: 39 m; decimalLatitude: 23.2046; decimalLongitude: 58.9692; georeferenceProtocol: label; **Identification:** identifiedBy: Mostafa R. Sharaf; dateIdentified: 2016; **Event:** samplingProtocol: Hand picking; eventDate: 2016-04-08; **Record Level:** language: en; collectionCode: KSMA; datasetName: Ants; ownerInstitutionCode: KSU; basisOfRecord: PreservedSpecimen
- **Type status:** Other material. **Occurrence:** recordedBy: Mostafa Sharaf; individualCount: 3; sex: worker; lifeStage: adult; **Taxon:** scientificName: Tapinoma melanocephalum; **Location:** country: Oman; locality: Ayn Hamran; verbatimElevation: 106 m; decimalLatitude: 17.09952; decimalLongitude: 54.28403; georeferenceProtocol: label; **Identification:** identifiedBy: Mostafa R. Sharaf; dateIdentified: 2017; **Event:** samplingProtocol: Sweeping; eventDate: 2017-11-20; **Record Level:** language: en; collectionCode: KSMA; datasetName: Ants; ownerInstitutionCode: KSU; basisOfRecord: PreservedSpecimen
- **Type status:** Other material. **Occurrence:** recordedBy: Mostafa Sharaf; individualCount: 10; sex: worker; lifeStage: adult; **Taxon:** scientificName: Tapinoma melanocephalum; **Location:** country: Oman; locality: Ayn Hamran; verbatimElevation: 56 m; decimalLatitude: 17.08631; decimalLongitude: 54.28043; georeferenceProtocol: label; **Identification:** identifiedBy: Mostafa R. Sharaf; dateIdentified: 2017; **Event:** samplingProtocol: Sifting try; eventDate: 2017-11-22; **Record Level:** language: en; collectionCode: KSMA; datasetName: Ants; ownerInstitutionCode: KSU; basisOfRecord: PreservedSpecimen
- **Type status:** Other material. **Occurrence:** recordedBy: Mostafa Sharaf; individualCount: 2; sex: worker; lifeStage: adult; **Taxon:** scientificName: Tapinoma melanocephalum; **Location:** country: Oman; locality: Ayn Sahlanot; verbatimElevation: 151 m; decimalLatitude: 17.14766; decimalLongitude: 54.17878; georeferenceProtocol: label; **Identification:** identifiedBy: Mostafa R. Sharaf; dateIdentified: 2017; **Event:** samplingProtocol: Malaise trap; eventDate: 2017-11-16; **Record Level:** language: en; collectionCode: KSMA; datasetName: Ants; ownerInstitutionCode: KSU; basisOfRecord: PreservedSpecimen
- **Type status:** Other material. **Occurrence:** recordedBy: Mostafa Sharaf; individualCount: 11; sex: worker; lifeStage: adult; **Taxon:** scientificName: Tapinoma melanocephalum; **Location:** country: Oman; locality: Dhalkout; verbatimElevation: 34 m; decimalLatitude: 16.70703; decimalLongitude: 53.25068; georeferenceProtocol: label; **Identification:** identifiedBy: Mostafa R. Sharaf; dateIdentified: 2017; **Event:** samplingProtocol: Sifting try; eventDate: 2017-11-19; **Record Level:** language: en; collectionCode: KSMA; datasetName: Ants; ownerInstitutionCode: KSU; basisOfRecord: PreservedSpecimen
- **Type status:** Other material. **Occurrence:** recordedBy: Mostafa Sharaf; individualCount: 7; sex: worker; lifeStage: adult; **Taxon:** scientificName: Tapinoma melanocephalum; **Location:** country: Oman; locality: Dhalkout; verbatimElevation: 43 m; decimalLatitude: 16.70492; decimalLongitude: 53.24453; georeferenceProtocol: label; **Identification:** identifiedBy: Mostafa R. Sharaf; dateIdentified: 2017; **Event:** samplingProtocol: Sifting try; eventDate: 2017-11-18; **Record Level:** language: en; collectionCode: KSMA; datasetName: Ants; ownerInstitutionCode: KSU; basisOfRecord: PreservedSpecimen
- **Type status:** Other material. **Occurrence:** recordedBy: Mostafa Sharaf; individualCount: 2; sex: worker; lifeStage: adult; **Taxon:** scientificName: Tapinoma melanocephalum; **Location:** country: Oman; locality: Dhalkout Rd., Aghbaroot Village; verbatimElevation: 1034 m; decimalLatitude: 16.79818; decimalLongitude: 53.55392; georeferenceProtocol: label; **Identification:** identifiedBy: Mostafa R. Sharaf; dateIdentified: 2017; **Event:** samplingProtocol: Sifting try; eventDate: 2017-11-18; **Record Level:** language: en; collectionCode: KSMA; datasetName: Ants; ownerInstitutionCode: KSU; basisOfRecord: PreservedSpecimen
- **Type status:** Other material. **Occurrence:** recordedBy: Mostafa Sharaf; individualCount: 3; sex: worker; lifeStage: adult; **Taxon:** scientificName: Tapinoma melanocephalum; **Location:** country: Oman; locality: El-Ebreien; verbatimElevation: 903 m; decimalLatitude: 23.14069; decimalLongitude: 57.31261; georeferenceProtocol: label; **Identification:** identifiedBy: Mostafa R. Sharaf; dateIdentified: 2017; **Event:** samplingProtocol: Hand picking; eventDate: 2017-01-21; **Record Level:** language: en; collectionCode: KSMA; datasetName: Ants; ownerInstitutionCode: KSU; basisOfRecord: PreservedSpecimen
- **Type status:** Other material. **Occurrence:** recordedBy: Mostafa Sharaf; individualCount: 8; sex: worker; lifeStage: adult; **Taxon:** scientificName: Tapinoma melanocephalum; **Location:** country: Oman; locality: Alraha Village; verbatimElevation: 74 m; decimalLatitude: 23.56665; decimalLongitude: 58.1763; georeferenceProtocol: label; **Identification:** identifiedBy: Mostafa R. Sharaf; dateIdentified: 2017; **Event:** samplingProtocol: Hand picking; eventDate: 2017-01-18; **Record Level:** language: en; collectionCode: KSMA; datasetName: Ants; ownerInstitutionCode: KSU; basisOfRecord: PreservedSpecimen
- **Type status:** Other material. **Occurrence:** recordedBy: Mostafa Sharaf; individualCount: 6; sex: worker; lifeStage: adult; **Taxon:** scientificName: Tapinoma melanocephalum; **Location:** country: Oman; locality: Muscat; verbatimElevation: 81 m; decimalLatitude: 23.6176; decimalLongitude: 58.49364; georeferenceProtocol: label; **Identification:** identifiedBy: Mostafa R. Sharaf; dateIdentified: 2016; **Event:** samplingProtocol: Hand picking; eventDate: 2016-04-07; **Record Level:** language: en; collectionCode: KSMA; datasetName: Ants; ownerInstitutionCode: KSU; basisOfRecord: PreservedSpecimen
- **Type status:** Other material. **Occurrence:** recordedBy: M. Gallagher; individualCount: 1; sex: worker; lifeStage: adult; **Taxon:** scientificName: Tapinoma melanocephalum; **Location:** country: Oman; locality: Salalah; decimalLatitude: 17.02; decimalLongitude: 54.08; georeferenceProtocol: label; **Identification:** identifiedBy: Cedric A. Collingwood; dateIdentified: 1988; **Event:** samplingProtocol: none specified; eventDate: 1905-06-07; **Record Level:** language: en; collectionCode: WMLC; datasetName: Ants; basisOfRecord: PreservedSpecimen
- **Type status:** Other material. **Occurrence:** recordedBy: M. Gallagher; individualCount: 1; sex: worker; lifeStage: adult; **Taxon:** scientificName: Tapinoma melanocephalum; **Location:** country: Oman; locality: Wahiba Sands; decimalLatitude: 21.33; decimalLongitude: 59.13; georeferenceProtocol: label; **Identification:** identifiedBy: Cedric A. Collingwood; dateIdentified: 1988; **Event:** samplingProtocol: none specified; eventDate: 1905-06-06; **Record Level:** language: en; collectionCode: WMLC; datasetName: Ants; basisOfRecord: PreservedSpecimen
- **Type status:** Other material. **Occurrence:** recordedBy: Mostafa Sharaf; individualCount: 5; sex: worker; lifeStage: adult; **Taxon:** scientificName: Tapinoma melanocephalum; **Location:** country: Oman; locality: Alkhoud Village; verbatimElevation: 63 m; decimalLatitude: 23.57154; decimalLongitude: 58.12166; georeferenceProtocol: label; **Identification:** identifiedBy: Mostafa R. Sharaf; dateIdentified: 2017; **Event:** samplingProtocol: Hand picking; eventDate: 2017-01-19; **Record Level:** language: en; collectionCode: KSMA; datasetName: Ants; ownerInstitutionCode: KSU; basisOfRecord: PreservedSpecimen
- **Type status:** Other material. **Occurrence:** recordedBy: Mostafa Sharaf; individualCount: 4; sex: worker; lifeStage: adult; **Taxon:** scientificName: Tapinoma melanocephalum; **Location:** country: Oman; locality: Fanga; verbatimElevation: 166 m; decimalLatitude: 23.45336; decimalLongitude: 58.11807; georeferenceProtocol: label; **Identification:** identifiedBy: Mostafa R. Sharaf; dateIdentified: 2017; **Event:** samplingProtocol: Hand picking; eventDate: 2017-01-20; **Record Level:** language: en; collectionCode: KSMA; datasetName: Ants; ownerInstitutionCode: KSU; basisOfRecord: PreservedSpecimen
- **Type status:** Other material. **Occurrence:** recordedBy: Mostafa Sharaf; individualCount: 3; sex: worker; lifeStage: adult; **Taxon:** scientificName: Tapinoma melanocephalum; **Location:** country: Oman; locality: Nakhl; verbatimElevation: 322 m; decimalLatitude: 23.38803; decimalLongitude: 57.82937; georeferenceProtocol: label; **Identification:** identifiedBy: Mostafa R. Sharaf; dateIdentified: 2016; **Event:** samplingProtocol: Hand picking; eventDate: 2016-04-02; **Record Level:** language: en; collectionCode: KSMA; datasetName: Ants; ownerInstitutionCode: KSU; basisOfRecord: PreservedSpecimen
- **Type status:** Other material. **Occurrence:** recordedBy: Mahmoud Abdel-Dayem; individualCount: 1; sex: worker; lifeStage: adult; **Taxon:** scientificName: Tapinoma melanocephalum; **Location:** country: Qatar; locality: Rawdet Rashed; decimalLatitude: 25.233433; decimalLongitude: 51.204767; georeferenceProtocol: label; **Identification:** identifiedBy: Mostafa R. Sharaf; dateIdentified: 2005; **Event:** samplingProtocol: Hand picking; eventDate: 2005-03-12; **Record Level:** language: en; collectionCode: KSMA; datasetName: Ants; ownerInstitutionCode: KSU; basisOfRecord: PreservedSpecimen
- **Type status:** Other material. **Occurrence:** recordedBy: Mostafa Sharaf; individualCount: 2; sex: worker; lifeStage: adult; **Taxon:** scientificName: Tapinoma melanocephalum; **Location:** country: Saudi Arabia; locality: Wadi Bagara; verbatimElevation: 436 m; decimalLatitude: 18.79287; decimalLongitude: 42.01857; georeferenceProtocol: label; **Identification:** identifiedBy: Mostafa R. Sharaf; dateIdentified: 2012; **Event:** samplingProtocol: Hand picking; eventDate: 2012-11-10; **Record Level:** language: en; collectionCode: KSMA; datasetName: Ants; ownerInstitutionCode: KSU; basisOfRecord: PreservedSpecimen
- **Type status:** Other material. **Occurrence:** recordedBy: James Wetterer; individualCount: 1; sex: worker; lifeStage: adult; **Taxon:** scientificName: Tapinoma melanocephalum; **Location:** country: United Arab Emirates; locality: Palm Deira; decimalLatitude: 25.276; decimalLongitude: 55.3; georeferenceProtocol: label; **Identification:** identifiedBy: James K. Wetterer; dateIdentified: 2012; **Event:** samplingProtocol: none specified; eventDate: 2012-05-16; **Record Level:** language: en; collectionCode: JWPC; datasetName: Ants; basisOfRecord: PreservedSpecimen
- **Type status:** Other material. **Occurrence:** recordedBy: James Wetterer; individualCount: 1; sex: worker; lifeStage: adult; **Taxon:** scientificName: Tapinoma melanocephalum; **Location:** country: United Arab Emirates; locality: Al-Jubail; decimalLatitude: 25.351; decimalLongitude: 55.382; georeferenceProtocol: label; **Identification:** identifiedBy: James K. Wetterer; dateIdentified: 2012; **Event:** samplingProtocol: none specified; eventDate: 2012-05-16; **Record Level:** language: en; collectionCode: JWPC; datasetName: Ants; basisOfRecord: PreservedSpecimen
- **Type status:** Other material. **Occurrence:** recordedBy: Mostafa Sharaf; individualCount: 9; sex: worker; lifeStage: adult; **Taxon:** scientificName: Tapinoma melanocephalum; **Location:** country: Yemen; locality: Dehejamo; verbatimElevation: 563 m; decimalLatitude: 12.59049; decimalLongitude: 54.05205; georeferenceProtocol: label; **Identification:** identifiedBy: Mostafa R. Sharaf; dateIdentified: 2014; **Event:** samplingProtocol: none specified; eventDate: 2014-04-22; **Record Level:** language: en; collectionCode: KSMA; datasetName: Ants; ownerInstitutionCode: KSU; basisOfRecord: PreservedSpecimen
- **Type status:** Other material. **Occurrence:** recordedBy: Mostafa Sharaf; individualCount: 6; sex: worker; lifeStage: adult; **Taxon:** scientificName: Tapinoma melanocephalum; **Location:** country: Yemen; locality: Dixam, Wadi Zereg; verbatimElevation: 279 m; decimalLatitude: 12.46868; decimalLongitude: 54.01091; georeferenceProtocol: label; **Identification:** identifiedBy: Mostafa R. Sharaf; dateIdentified: 2014; **Event:** samplingProtocol: Hand picking; eventDate: 2014-04-24; **Record Level:** language: en; collectionCode: KSMA; datasetName: Ants; ownerInstitutionCode: KSU; basisOfRecord: PreservedSpecimen
- **Type status:** Other material. **Occurrence:** recordedBy: Mostafa Sharaf; individualCount: 3; sex: worker; lifeStage: adult; **Taxon:** scientificName: Tapinoma melanocephalum; **Location:** country: Yemen; locality: Himihil Protectorate; verbatimElevation: 372 m; decimalLatitude: 12.57615; decimalLongitude: 54.30651; georeferenceProtocol: label; **Identification:** identifiedBy: Mostafa R. Sharaf; dateIdentified: 2014; **Event:** samplingProtocol: Hand picking; eventDate: 2014-04-23; **Record Level:** language: en; collectionCode: KSMA; datasetName: Ants; ownerInstitutionCode: KSU; basisOfRecord: PreservedSpecimen
- **Type status:** Other material. **Occurrence:** recordedBy: Mostafa Sharaf; individualCount: 2; sex: worker; lifeStage: adult; **Taxon:** scientificName: Tapinoma melanocephalum; **Location:** country: Yemen; locality: Lehanoh; verbatimElevation: 931 m; decimalLatitude: 12.57583; decimalLongitude: 54.04836; georeferenceProtocol: label; **Identification:** identifiedBy: Mostafa R. Sharaf; dateIdentified: 2014; **Event:** samplingProtocol: Hand picking; eventDate: 2014-04-22; **Record Level:** language: en; collectionCode: KSMA; datasetName: Ants; ownerInstitutionCode: KSU; basisOfRecord: PreservedSpecimen

#### Diagnosis

**Worker.** This is an easily recognised species by its bicoloured body (Fig. 2a, b), with head and mesosoma pale brown to dark brown, antennae, maxillary palps and mandibles pale brown to yellow, gaster and legs pale yellow. Head in full-face view with scapes, when laid back from their insertions, surpass posterior margin of head (Fig. 2c); eyes located in front of mid-length of head (Fig. 2c). The size is small (1.20–1.90 mm) and mesosoma is without dorsal hairs.

##### Previous records

**KUWAIT**: Kuwait City, 29.37°N, 47.98°E (Collingwood and Agosti 1996). **OMAN**: Hawiyah, 22.39°N, 58.85°E (Collingwood 1988); Mintirib, 22.56°N, 58.03°E (Collingwood 1988); Salalah, 17.02°N, 54.08°E (Collingwood 1988); Umm Qishrib, (Collingwood 1988); Wahiba Sands, 21.33°N, 59.13°E (Collingwood 1985). **QATAR**: Rawdet Rashed, 25°14.006’N, 51°12.286’E (Sharaf et al. 2020b). **SA**: Hofuf (Collingwood 1985); Al Qatif, (Collingwood 1985). **UAE**: Abu Dhabi, Al-Ain, 24.13°N, 55.80°E (Collingwood et al. 2011); Abu Dhabi, Al-Ajban, 24.56°N, 55.34°E (Collingwood et al. 2011); Sharjah, Sharjah Desert Park, 25.28°N, 55.70°E (Collingwood et al. 2011). **YEMEN**: Aden Chalet, 12.83°N, 45.00°E (Collingwood and Agosti 1996); Socotra Is., Hoq, 12.60°N, 54.35°E (Collingwood et al. 2004); Socotra Is., Wadi Daneghan, 12.62°N, 54.07°E (Collingwood et al. 2004); Socotra Is., Goeeh, 12.53°N, 54.17°E (Collingwood et al. 2004); Socotra Is., Farmihin, 12.53°N, 53.93°E (Collingwood et al. 2004); Socotra Is., Dirhashas, 12.53°N, 53.86°E (Collingwood et al. 2004); Socotra Is., Homhil, 12.57°N, 54.32°E (Collingwood et al. 2004); Lahj, 13.06°N, 44.88°E (Collingwood and van Harten 2001); Al Kowd, 13.09°N, 45.37°E (Collingwood and van Harten 2001); Mayfa'ah, 14.33°N, 46.00°E (Collingwood and van Harten 2001).

#### Distribution

*Tapinoma melanocephalum* is a widely distributed invasive species that is spread by human commerce especially in the Tropics and Subtropics of the Old and New Worlds (Wetterer 2009) with an unknown native range. The species is widely spread in several countries of the Arabian Peninsula, including SA and Oman (Collingwood 1985), Yemen (Collingwood and Agosti 1996), UAE (Collingwood et al. 1997), the Socotra Archipelago (Sharaf et al. 2017) and, recently, it has been reported from Qatar (Sharaf et al. 2020b).

#### Ecology

The species inhabits a broad range of habitats worldwide (Sharaf et al. 2017), including both humid and dry soil of wild and urban localities, in homes, restaurants, hospitals, well-heated buildings and greenhouses (Wetterer 2009), under bark and stones, in leaf litter and sometimes nests are built in walls and potted plants indoors (Smith and Whitman 1992, Ellison et al. 2012), in moist soil of lawn and grasses and under debris (Oster and Wilson 1978) or in opportunistic places (Hölldobler and Wilson 1990). In the Socotra Archipelago (Yemen), the species was observed foraging on a tree located on a mountainside next to a stream drainage where the soil was moist and the area was dominated by the ponerine ant, *Brachyponera sennaarensis* (Mayr, 1862) and *Adiantum capillus-veneris* L. (Pteridaceae) (Sharaf et al. 2017). It also inhabits localities rich in organic matter of animal faeces in date palm plantations (*Phoenix dactylifera* L.) (Sharaf et al. 2017). Workers attend honeydew-excreting insects for honeydew (Venkataramaiah and Rehman 1989) and also feed on both dead and live insects and when disturbed workers are running erratically and rapidly. (Smith 1965).

### Tapinoma simrothi

Krausse, 1911

58311883-F660-5751-9D01-B39D7A680761

https://www.gbif.org/species/1316856

Tapinoma erraticum var. simrothi Krausse, 1911a: 18 (w.) Italy (Sardinia). Palearctic.
Tapinoma erraticum var. simrothi Krausse, 1911a: 18 (w.) Italy (Sardinia). Palearctic.

#### Materials

- **Type status:** Other material. **Occurrence:** recordedBy: S. Salman; individualCount: 9; sex: worker; lifeStage: adult; **Taxon:** scientificName: Tapinoma simrothi; **Location:** country: Saudi Arabia; locality: Khobar, Prince Sultan Palace; verbatimElevation: 8 m; decimalLatitude: 26.34625; decimalLongitude: 50.2177; georeferenceProtocol: label; **Identification:** identifiedBy: Mostafa R. Sharaf; dateIdentified: 2013; **Event:** samplingProtocol: Hand picking; eventDate: 2013-12-20; **Record Level:** language: en; collectionCode: KSMA; datasetName: Ants; ownerInstitutionCode: KSU; basisOfRecord: PreservedSpecimen
- **Type status:** Other material. **Occurrence:** recordedBy: S. Salman; individualCount: 5; sex: worker; lifeStage: adult; **Taxon:** scientificName: Tapinoma simrothi; **Location:** country: Saudi Arabia; locality: Qatif, Ar Rimal; decimalLatitude: 26.49865; decimalLongitude: 50.00488; georeferenceProtocol: label; **Identification:** identifiedBy: Mostafa R. Sharaf; dateIdentified: 2013; **Event:** samplingProtocol: Hand picking; eventDate: 2013-12-17; **Record Level:** language: en; collectionCode: KSMA; datasetName: Ants; ownerInstitutionCode: KSU; basisOfRecord: PreservedSpecimen
- **Type status:** Other material. **Occurrence:** recordedBy: M.R. Sharaf; individualCount: 9; sex: worker; lifeStage: adult; **Taxon:** scientificName: Tapinoma simrothi; **Location:** country: Saudi Arabia; locality: Buraydah; verbatimElevation: 633 m; decimalLatitude: 26.2159; decimalLongitude: 44.041383; georeferenceProtocol: label; **Identification:** identifiedBy: Mostafa R. Sharaf; dateIdentified: 2011; **Event:** samplingProtocol: Hand picking; eventDate: 2011-09-17; **Record Level:** language: en; collectionCode: KSMA; datasetName: Ants; ownerInstitutionCode: KSU; basisOfRecord: PreservedSpecimen
- **Type status:** Other material. **Occurrence:** recordedBy: S. Salman; individualCount: 1; sex: worker; lifeStage: adult; **Taxon:** scientificName: Tapinoma simrothi; **Location:** country: Saudi Arabia; locality: Afif; verbatimElevation: 1052 m; decimalLatitude: 23.90005; decimalLongitude: 42.88075; georeferenceProtocol: label; **Identification:** identifiedBy: Mostafa R. Sharaf; dateIdentified: 2015; **Event:** samplingProtocol: Hand picking; eventDate: 2015-01-17; **Record Level:** language: en; collectionCode: KSMA; datasetName: Ants; ownerInstitutionCode: KSU; basisOfRecord: PreservedSpecimen
- **Type status:** Other material. **Occurrence:** recordedBy: S. Salman; individualCount: 3; sex: worker; lifeStage: adult; **Taxon:** scientificName: Tapinoma simrothi; **Location:** country: Saudi Arabia; locality: Al Aflag, Layla; verbatimElevation: 562 m; decimalLatitude: 22.23719; decimalLongitude: 46.69951; georeferenceProtocol: label; **Identification:** identifiedBy: Mostafa R. Sharaf; dateIdentified: 2014; **Event:** samplingProtocol: Hand picking; eventDate: 2014-01-21; **Record Level:** language: en; collectionCode: KSMA; datasetName: Ants; ownerInstitutionCode: KSU; basisOfRecord: PreservedSpecimen
- **Type status:** Other material. **Occurrence:** recordedBy: S. Salman; individualCount: 2; sex: worker; lifeStage: adult; **Taxon:** scientificName: Tapinoma simrothi; **Location:** country: Saudi Arabia; locality: Al Ghat; verbatimElevation: 670 m; decimalLatitude: 26.02495; decimalLongitude: 44.9365; georeferenceProtocol: label; **Identification:** identifiedBy: Mostafa R. Sharaf; dateIdentified: 2014; **Event:** samplingProtocol: Hand picking; eventDate: 2014-06-07; **Record Level:** language: en; collectionCode: KSMA; datasetName: Ants; ownerInstitutionCode: KSU; basisOfRecord: PreservedSpecimen
- **Type status:** Other material. **Occurrence:** recordedBy: S. Salman; individualCount: 3; sex: worker; lifeStage: adult; **Taxon:** scientificName: Tapinoma simrothi; **Location:** country: Saudi Arabia; locality: Al Ghat; verbatimElevation: 679 m; decimalLatitude: 26.02219; decimalLongitude: 44.9876; georeferenceProtocol: label; **Identification:** identifiedBy: Mostafa R. Sharaf; dateIdentified: 2014; **Event:** samplingProtocol: Hand picking; eventDate: 2014-09-13; **Record Level:** language: en; collectionCode: KSMA; datasetName: Ants; ownerInstitutionCode: KSU; basisOfRecord: PreservedSpecimen
- **Type status:** Other material. **Occurrence:** recordedBy: S. Salman; individualCount: 3; sex: worker; lifeStage: adult; **Taxon:** scientificName: Tapinoma simrothi; **Location:** country: Saudi Arabia; locality: Al Ghat; verbatimElevation: 653 m; decimalLatitude: 26.06582; decimalLongitude: 44.91929; georeferenceProtocol: label; **Identification:** identifiedBy: Mostafa R. Sharaf; dateIdentified: 2015; **Event:** samplingProtocol: Hand picking; eventDate: 2015-10-31; **Record Level:** language: en; collectionCode: KSMA; datasetName: Ants; ownerInstitutionCode: KSU; basisOfRecord: PreservedSpecimen
- **Type status:** Other material. **Occurrence:** recordedBy: M.R. Sharaf; individualCount: 6; sex: worker; lifeStage: adult; **Taxon:** scientificName: Tapinoma simrothi; **Location:** country: Saudi Arabia; locality: Al Hayer; verbatimElevation: 587 m; decimalLatitude: 24.55755; decimalLongitude: 46.74115; georeferenceProtocol: label; **Identification:** identifiedBy: Mostafa R. Sharaf; dateIdentified: 2013; **Event:** samplingProtocol: Hand picking; eventDate: 2013-10-09; **Record Level:** language: en; collectionCode: KSMA; datasetName: Ants; ownerInstitutionCode: KSU; basisOfRecord: PreservedSpecimen
- **Type status:** Other material. **Occurrence:** recordedBy: S. Salman; individualCount: 4; sex: worker; lifeStage: adult; **Taxon:** scientificName: Tapinoma simrothi; **Location:** country: Saudi Arabia; locality: Al Hayer; verbatimElevation: 467 m; decimalLatitude: 24.54645; decimalLongitude: 46.74156; georeferenceProtocol: label; **Identification:** identifiedBy: Mostafa R. Sharaf; dateIdentified: 2014; **Event:** samplingProtocol: Hand picking; eventDate: 2014-04-11; **Record Level:** language: en; collectionCode: KSMA; datasetName: Ants; ownerInstitutionCode: KSU; basisOfRecord: PreservedSpecimen
- **Type status:** Other material. **Occurrence:** recordedBy: S. Salman; individualCount: 3; sex: worker; lifeStage: adult; **Taxon:** scientificName: Tapinoma simrothi; **Location:** country: Saudi Arabia; locality: Al Kharj; verbatimElevation: 453 m; decimalLatitude: 24.1436; decimalLongitude: 47.24252; georeferenceProtocol: label; **Identification:** identifiedBy: Mostafa R. Sharaf; dateIdentified: 2014; **Event:** samplingProtocol: Hand picking; eventDate: 2014-01-19; **Record Level:** language: en; collectionCode: KSMA; datasetName: Ants; ownerInstitutionCode: KSU; basisOfRecord: PreservedSpecimen
- **Type status:** Other material. **Occurrence:** recordedBy: S. Salman; individualCount: 9; sex: worker; lifeStage: adult; **Taxon:** scientificName: Tapinoma simrothi; **Location:** country: Saudi Arabia; locality: Al Kharj; verbatimElevation: 451 m; decimalLatitude: 24.29615; decimalLongitude: 47.15553; georeferenceProtocol: label; **Identification:** identifiedBy: Mostafa R. Sharaf; dateIdentified: 2014; **Event:** samplingProtocol: Hand picking; eventDate: 2014-09-08; **Record Level:** language: en; collectionCode: KSMA; datasetName: Ants; ownerInstitutionCode: KSU; basisOfRecord: PreservedSpecimen
- **Type status:** Other material. **Occurrence:** recordedBy: M.R. Sharaf; individualCount: 17; sex: worker; lifeStage: adult; **Taxon:** scientificName: Tapinoma simrothi; **Location:** country: Saudi Arabia; locality: Ammariya; verbatimElevation: 633 m; decimalLatitude: 23.8333; decimalLongitude: 45; georeferenceProtocol: label; **Identification:** identifiedBy: Mostafa R. Sharaf; dateIdentified: 2008; **Event:** samplingProtocol: Hand picking; eventDate: 2008-07-07; **Record Level:** language: en; collectionCode: KSMA; datasetName: Ants; ownerInstitutionCode: KSU; basisOfRecord: PreservedSpecimen
- **Type status:** Other material. **Occurrence:** recordedBy: S. Salman; individualCount: 5; sex: worker; lifeStage: adult; **Taxon:** scientificName: Tapinoma simrothi; **Location:** country: Saudi Arabia; locality: Dirab; verbatimElevation: 604 m; decimalLatitude: 24.41867; decimalLongitude: 46.65408; georeferenceProtocol: label; **Identification:** identifiedBy: Mostafa R. Sharaf; dateIdentified: 2014; **Event:** samplingProtocol: Hand picking; eventDate: 2014-09-18; **Record Level:** language: en; collectionCode: KSMA; datasetName: Ants; ownerInstitutionCode: KSU; basisOfRecord: PreservedSpecimen
- **Type status:** Other material. **Occurrence:** recordedBy: S. Salman; individualCount: 1; sex: worker; lifeStage: adult; **Taxon:** scientificName: Tapinoma simrothi; **Location:** country: Saudi Arabia; locality: Hawtat Bani Tamim; verbatimElevation: 582 m; decimalLatitude: 23.45943; decimalLongitude: 46.81895; georeferenceProtocol: label; **Identification:** identifiedBy: Mostafa R. Sharaf; dateIdentified: 2015; **Event:** samplingProtocol: Hand picking; eventDate: 2015-02-19; **Record Level:** language: en; collectionCode: KSMA; datasetName: Ants; ownerInstitutionCode: KSU; basisOfRecord: PreservedSpecimen
- **Type status:** Other material. **Occurrence:** recordedBy: S. Salman; individualCount: 2; sex: worker; lifeStage: adult; **Taxon:** scientificName: Tapinoma simrothi; **Location:** country: Saudi Arabia; locality: Hawtat Sudayr; verbatimElevation: 732 m; decimalLatitude: 25.59162; decimalLongitude: 45.61245; georeferenceProtocol: label; **Identification:** identifiedBy: Mostafa R. Sharaf; dateIdentified: 2015; **Event:** samplingProtocol: Hand picking; eventDate: 2015-01-31; **Record Level:** language: en; collectionCode: KSMA; datasetName: Ants; ownerInstitutionCode: KSU; basisOfRecord: PreservedSpecimen
- **Type status:** Other material. **Occurrence:** recordedBy: M.R. Sharaf; individualCount: 9; sex: worker; lifeStage: adult; **Taxon:** scientificName: Tapinoma simrothi; **Location:** country: Saudi Arabia; locality: Huraymila; verbatimElevation: 785 m; decimalLatitude: 25.127861; decimalLongitude: 46.088278; georeferenceProtocol: label; **Identification:** identifiedBy: Mostafa R. Sharaf; dateIdentified: 2011; **Event:** samplingProtocol: Hand picking; eventDate: 2011-02-07; **Record Level:** language: en; collectionCode: KSMA; datasetName: Ants; ownerInstitutionCode: KSU; basisOfRecord: PreservedSpecimen
- **Type status:** Other material. **Occurrence:** recordedBy: S. Salman; individualCount: 4; sex: worker; lifeStage: adult; **Taxon:** scientificName: Tapinoma simrothi; **Location:** country: Saudi Arabia; locality: Huraymila; verbatimElevation: 791 m; decimalLatitude: 25.12799; decimalLongitude: 46.11783; georeferenceProtocol: label; **Identification:** identifiedBy: Mostafa R. Sharaf; dateIdentified: 2014; **Event:** samplingProtocol: Hand picking; eventDate: 2014-02-22; **Record Level:** language: en; collectionCode: KSMA; datasetName: Ants; ownerInstitutionCode: KSU; basisOfRecord: PreservedSpecimen
- **Type status:** Other material. **Occurrence:** recordedBy: S. Salman; individualCount: 9; sex: worker; lifeStage: adult; **Taxon:** scientificName: Tapinoma simrothi; **Location:** country: Saudi Arabia; locality: Huraymila; verbatimElevation: 785 m; decimalLatitude: 25.09757; decimalLongitude: 46.10106; georeferenceProtocol: label; **Identification:** identifiedBy: Mostafa R. Sharaf; dateIdentified: 2014; **Event:** samplingProtocol: Hand picking; eventDate: 2014-02-22; **Record Level:** language: en; collectionCode: KSMA; datasetName: Ants; ownerInstitutionCode: KSU; basisOfRecord: PreservedSpecimen
- **Type status:** Other material. **Occurrence:** recordedBy: S. Salman; individualCount: 5; sex: worker; lifeStage: adult; **Taxon:** scientificName: Tapinoma simrothi; **Location:** country: Saudi Arabia; locality: Huraymila; verbatimElevation: 786 m; decimalLatitude: 25.12037; decimalLongitude: 46.12265; georeferenceProtocol: label; **Identification:** identifiedBy: Mostafa R. Sharaf; dateIdentified: 2014; **Event:** samplingProtocol: Hand picking; eventDate: 2014-02-22; **Record Level:** language: en; collectionCode: KSMA; datasetName: Ants; ownerInstitutionCode: KSU; basisOfRecord: PreservedSpecimen
- **Type status:** Other material. **Occurrence:** recordedBy: S. Salman; individualCount: 4; sex: worker; lifeStage: adult; **Taxon:** scientificName: Tapinoma simrothi; **Location:** country: Saudi Arabia; locality: Huraymila; verbatimElevation: 771 m; decimalLatitude: 25.12636; decimalLongitude: 46.15782; georeferenceProtocol: label; **Identification:** identifiedBy: Mostafa R. Sharaf; dateIdentified: 2014; **Event:** samplingProtocol: Hand picking; eventDate: 2014-02-22; **Record Level:** language: en; collectionCode: KSMA; datasetName: Ants; ownerInstitutionCode: KSU; basisOfRecord: PreservedSpecimen
- **Type status:** Other material. **Occurrence:** recordedBy: S. Salman; individualCount: 2; sex: worker; lifeStage: adult; **Taxon:** scientificName: Tapinoma simrothi; **Location:** country: Saudi Arabia; locality: Huraymila; verbatimElevation: 801 m; decimalLatitude: 25.09358; decimalLongitude: 46.08141; georeferenceProtocol: label; **Identification:** identifiedBy: Mostafa R. Sharaf; dateIdentified: 2014; **Event:** samplingProtocol: Hand picking; eventDate: 2014-02-22; **Record Level:** language: en; collectionCode: KSMA; datasetName: Ants; ownerInstitutionCode: KSU; basisOfRecord: PreservedSpecimen
- **Type status:** Other material. **Occurrence:** recordedBy: M.R. Sharaf; individualCount: 3; sex: worker; lifeStage: adult; **Taxon:** scientificName: Tapinoma simrothi; **Location:** country: Saudi Arabia; locality: King Saud University Campus; verbatimElevation: 687 m; decimalLatitude: 24.716667; decimalLongitude: 46.616667; georeferenceProtocol: label; **Identification:** identifiedBy: Mostafa R. Sharaf; dateIdentified: 2009; **Event:** samplingProtocol: Hand picking; eventDate: 2009-10-19; **Record Level:** language: en; collectionCode: KSMA; datasetName: Ants; ownerInstitutionCode: KSU; basisOfRecord: PreservedSpecimen
- **Type status:** Other material. **Occurrence:** recordedBy: S. Salman; individualCount: 18; sex: worker; lifeStage: adult; **Taxon:** scientificName: Tapinoma simrothi; **Location:** country: Saudi Arabia; locality: King Saud University Campus; verbatimElevation: 687 m; decimalLatitude: 24.716667; decimalLongitude: 46.616667; georeferenceProtocol: label; **Identification:** identifiedBy: Mostafa R. Sharaf; dateIdentified: 2013; **Event:** samplingProtocol: Hand picking; eventDate: 2013-03-15; **Record Level:** language: en; collectionCode: KSMA; datasetName: Ants; ownerInstitutionCode: KSU; basisOfRecord: PreservedSpecimen
- **Type status:** Other material. **Occurrence:** recordedBy: S. Salman; individualCount: 28; sex: worker; lifeStage: adult; **Taxon:** scientificName: Tapinoma simrothi; **Location:** country: Saudi Arabia; locality: King Saud University Campus; verbatimElevation: 671 m; decimalLatitude: 24.721683; decimalLongitude: 46.622083; georeferenceProtocol: label; **Identification:** identifiedBy: Mostafa R. Sharaf; dateIdentified: 2013; **Event:** samplingProtocol: Hand picking; eventDate: 2013-03-11; **Record Level:** language: en; collectionCode: KSMA; datasetName: Ants; ownerInstitutionCode: KSU; basisOfRecord: PreservedSpecimen
- **Type status:** Other material. **Occurrence:** recordedBy: S. Salman; individualCount: 8; sex: worker; lifeStage: adult; **Taxon:** scientificName: Tapinoma simrothi; **Location:** country: Saudi Arabia; locality: King Saud University Campus; verbatimElevation: 681 m; decimalLatitude: 24.72185; decimalLongitude: 46.621183; georeferenceProtocol: label; **Identification:** identifiedBy: Mostafa R. Sharaf; dateIdentified: 2013; **Event:** samplingProtocol: Hand picking; eventDate: 2013-03-07; **Record Level:** language: en; collectionCode: KSMA; datasetName: Ants; ownerInstitutionCode: KSU; basisOfRecord: PreservedSpecimen
- **Type status:** Other material. **Occurrence:** recordedBy: S. Salman; individualCount: 4; sex: worker; lifeStage: adult; **Taxon:** scientificName: Tapinoma simrothi; **Location:** country: Saudi Arabia; locality: Majmaa; verbatimElevation: 722 m; decimalLatitude: 25.91771; decimalLongitude: 45.33556; georeferenceProtocol: label; **Identification:** identifiedBy: Mostafa R. Sharaf; dateIdentified: 2014; **Event:** samplingProtocol: Hand picking; eventDate: 2014-06-05; **Record Level:** language: en; collectionCode: KSMA; datasetName: Ants; ownerInstitutionCode: KSU; basisOfRecord: PreservedSpecimen
- **Type status:** Other material. **Occurrence:** recordedBy: S. Salman; individualCount: 3; sex: worker; lifeStage: adult; **Taxon:** scientificName: Tapinoma simrothi; **Location:** country: Saudi Arabia; locality: Majmaa; verbatimElevation: 746 m; decimalLatitude: 25.93117; decimalLongitude: 45.35802; georeferenceProtocol: label; **Identification:** identifiedBy: Mostafa R. Sharaf; dateIdentified: 2014; **Event:** samplingProtocol: Hand picking; eventDate: 2014-09-13; **Record Level:** language: en; collectionCode: KSMA; datasetName: Ants; ownerInstitutionCode: KSU; basisOfRecord: PreservedSpecimen
- **Type status:** Other material. **Occurrence:** recordedBy: S. Salman; individualCount: 3; sex: worker; lifeStage: adult; **Taxon:** scientificName: Tapinoma simrothi; **Location:** country: Saudi Arabia; locality: Malham; verbatimElevation: 711 m; decimalLatitude: 25.15427; decimalLongitude: 46.28214; georeferenceProtocol: label; **Identification:** identifiedBy: Mostafa R. Sharaf; dateIdentified: 2014; **Event:** samplingProtocol: Hand picking; eventDate: 2014-09-15; **Record Level:** language: en; collectionCode: KSMA; datasetName: Ants; ownerInstitutionCode: KSU; basisOfRecord: PreservedSpecimen
- **Type status:** Other material. **Occurrence:** recordedBy: S. Salman; individualCount: 9; sex: worker; lifeStage: adult; **Taxon:** scientificName: Tapinoma simrothi; **Location:** country: Saudi Arabia; locality: Mozahmyia; verbatimElevation: 677 m; decimalLatitude: 24.45125; decimalLongitude: 46.20202; georeferenceProtocol: label; **Identification:** identifiedBy: Mostafa R. Sharaf; dateIdentified: 2014; **Event:** samplingProtocol: Hand picking; eventDate: 2014-01-25; **Record Level:** language: en; collectionCode: KSMA; datasetName: Ants; ownerInstitutionCode: KSU; basisOfRecord: PreservedSpecimen
- **Type status:** Other material. **Occurrence:** recordedBy: S. Salman; individualCount: 2; sex: worker; lifeStage: adult; **Taxon:** scientificName: Tapinoma simrothi; **Location:** country: Saudi Arabia; locality: Mozahmyia; verbatimElevation: 641 m; decimalLatitude: 24.46553; decimalLongitude: 46.2201; georeferenceProtocol: label; **Identification:** identifiedBy: Mostafa R. Sharaf; dateIdentified: 2014; **Event:** samplingProtocol: Hand picking; eventDate: 2014-01-25; **Record Level:** language: en; collectionCode: KSMA; datasetName: Ants; ownerInstitutionCode: KSU; basisOfRecord: PreservedSpecimen
- **Type status:** Other material. **Occurrence:** recordedBy: S. Salman; individualCount: 2; sex: worker; lifeStage: adult; **Taxon:** scientificName: Tapinoma simrothi; **Location:** country: Saudi Arabia; locality: Mozahmyia; verbatimElevation: 648 m; decimalLatitude: 24.48388; decimalLongitude: 46.26317; georeferenceProtocol: label; **Identification:** identifiedBy: Mostafa R. Sharaf; dateIdentified: 2014; **Event:** samplingProtocol: Hand picking; eventDate: 2014-01-25; **Record Level:** language: en; collectionCode: KSMA; datasetName: Ants; ownerInstitutionCode: KSU; basisOfRecord: PreservedSpecimen
- **Type status:** Other material. **Occurrence:** recordedBy: S. Salman; individualCount: 6; sex: worker; lifeStage: adult; **Taxon:** scientificName: Tapinoma simrothi; **Location:** country: Saudi Arabia; locality: Na'jan; verbatimElevation: 513 m; decimalLatitude: 24.06858; decimalLongitude: 47.1725; georeferenceProtocol: label; **Identification:** identifiedBy: Mostafa R. Sharaf; dateIdentified: 2014; **Event:** samplingProtocol: Hand picking; eventDate: 2014-12-13; **Record Level:** language: en; collectionCode: KSMA; datasetName: Ants; ownerInstitutionCode: KSU; basisOfRecord: PreservedSpecimen
- **Type status:** Other material. **Occurrence:** recordedBy: M.R. Sharaf; individualCount: 2; sex: worker; lifeStage: adult; **Taxon:** scientificName: Tapinoma simrothi; **Location:** country: Saudi Arabia; locality: Oyaina; verbatimElevation: 749 m; decimalLatitude: 24.90665; decimalLongitude: 46.389917; georeferenceProtocol: label; **Identification:** identifiedBy: Mostafa R. Sharaf; dateIdentified: 2010; **Event:** samplingProtocol: Hand picking; eventDate: 2010-04-28; **Record Level:** language: en; collectionCode: KSMA; datasetName: Ants; ownerInstitutionCode: KSU; basisOfRecord: PreservedSpecimen
- **Type status:** Other material. **Occurrence:** recordedBy: S. Salman; individualCount: 15; sex: worker; lifeStage: adult; **Taxon:** scientificName: Tapinoma simrothi; **Location:** country: Saudi Arabia; locality: Riyadh City, Al Emam University; verbatimElevation: 650 m; decimalLatitude: 24.81658; decimalLongitude: 46.71162; georeferenceProtocol: label; **Identification:** identifiedBy: Mostafa R. Sharaf; dateIdentified: 2014; **Event:** samplingProtocol: Hand picking; eventDate: 2014-09-08; **Record Level:** language: en; collectionCode: KSMA; datasetName: Ants; ownerInstitutionCode: KSU; basisOfRecord: PreservedSpecimen
- **Type status:** Other material. **Occurrence:** recordedBy: S. Salman; individualCount: 5; sex: worker; lifeStage: adult; **Taxon:** scientificName: Tapinoma simrothi; **Location:** country: Saudi Arabia; locality: Shaqra; verbatimElevation: 728 m; decimalLatitude: 25.26655; decimalLongitude: 45.26779; georeferenceProtocol: label; **Identification:** identifiedBy: Mostafa R. Sharaf; dateIdentified: 2014; **Event:** samplingProtocol: Hand picking; eventDate: 2014-05-30; **Record Level:** language: en; collectionCode: KSMA; datasetName: Ants; ownerInstitutionCode: KSU; basisOfRecord: PreservedSpecimen
- **Type status:** Other material. **Occurrence:** recordedBy: S. Salman; individualCount: 1; sex: worker; lifeStage: adult; **Taxon:** scientificName: Tapinoma simrothi; **Location:** country: Saudi Arabia; locality: Shaqra; verbatimElevation: 891 m; decimalLatitude: 25.12131; decimalLongitude: 45.48365; georeferenceProtocol: label; **Identification:** identifiedBy: Mostafa R. Sharaf; dateIdentified: 2015; **Event:** samplingProtocol: Hand picking; eventDate: 2015-01-23; **Record Level:** language: en; collectionCode: KSMA; datasetName: Ants; ownerInstitutionCode: KSU; basisOfRecord: PreservedSpecimen
- **Type status:** Other material. **Occurrence:** recordedBy: S. Salman; individualCount: 1; sex: worker; lifeStage: adult; **Taxon:** scientificName: Tapinoma simrothi; **Location:** country: Saudi Arabia; locality: Tebrak; verbatimElevation: 724 m; decimalLatitude: 24.32406; decimalLongitude: 45.47128; georeferenceProtocol: label; **Identification:** identifiedBy: Mostafa R. Sharaf; dateIdentified: 2014; **Event:** samplingProtocol: Hand picking; eventDate: 2014-11-29; **Record Level:** language: en; collectionCode: KSMA; datasetName: Ants; ownerInstitutionCode: KSU; basisOfRecord: PreservedSpecimen
- **Type status:** Other material. **Occurrence:** recordedBy: S. Salman; individualCount: 1; sex: worker; lifeStage: adult; **Taxon:** scientificName: Tapinoma simrothi; **Location:** country: Saudi Arabia; locality: Thadiq; verbatimElevation: 735 m; decimalLatitude: 25.2936; decimalLongitude: 45.87102; georeferenceProtocol: label; **Identification:** identifiedBy: Mostafa R. Sharaf; dateIdentified: 2014; **Event:** samplingProtocol: Hand picking; eventDate: 2014-04-26; **Record Level:** language: en; collectionCode: KSMA; datasetName: Ants; ownerInstitutionCode: KSU; basisOfRecord: PreservedSpecimen
- **Type status:** Other material. **Occurrence:** recordedBy: S. Salman; individualCount: 6; sex: worker; lifeStage: adult; **Taxon:** scientificName: Tapinoma simrothi; **Location:** country: Saudi Arabia; locality: Wadi El Dawaser; verbatimElevation: 706 m; decimalLatitude: 20.49008; decimalLongitude: 44.76018; georeferenceProtocol: label; **Identification:** identifiedBy: Mostafa R. Sharaf; dateIdentified: 2014; **Event:** samplingProtocol: Hand picking; eventDate: 2014-01-22; **Record Level:** language: en; collectionCode: KSMA; datasetName: Ants; ownerInstitutionCode: KSU; basisOfRecord: PreservedSpecimen
- **Type status:** Other material. **Occurrence:** recordedBy: S. Salman; individualCount: 5; sex: worker; lifeStage: adult; **Taxon:** scientificName: Tapinoma simrothi; **Location:** country: Saudi Arabia; locality: Wadi El Dawaser; verbatimElevation: 686 m; decimalLatitude: 22.7774; decimalLongitude: 44.78624; georeferenceProtocol: label; **Identification:** identifiedBy: Mostafa R. Sharaf; dateIdentified: 2015; **Event:** samplingProtocol: Hand picking; eventDate: 2015-02-22; **Record Level:** language: en; collectionCode: KSMA; datasetName: Ants; ownerInstitutionCode: KSU; basisOfRecord: PreservedSpecimen
- **Type status:** Other material. **Occurrence:** recordedBy: S. Salman; individualCount: 3; sex: worker; lifeStage: adult; **Taxon:** scientificName: Tapinoma simrothi; **Location:** country: Saudi Arabia; locality: Wadi El Dawaser; verbatimElevation: 694 m; decimalLatitude: 22.47942; decimalLongitude: 44.78839; georeferenceProtocol: label; **Identification:** identifiedBy: Mostafa R. Sharaf; dateIdentified: 2015; **Event:** samplingProtocol: Hand picking; eventDate: 2015-02-22; **Record Level:** language: en; collectionCode: KSMA; datasetName: Ants; ownerInstitutionCode: KSU; basisOfRecord: PreservedSpecimen
- **Type status:** Other material. **Occurrence:** recordedBy: M.R. Sharaf; individualCount: 6; sex: worker; lifeStage: adult; **Taxon:** scientificName: Tapinoma simrothi; **Location:** country: Saudi Arabia; locality: Wadi Hanifa; verbatimElevation: 633 m; decimalLatitude: 24.658983; decimalLongitude: 46.60345; georeferenceProtocol: label; **Identification:** identifiedBy: Mostafa R. Sharaf; dateIdentified: 2010; **Event:** samplingProtocol: Hand picking; eventDate: 2010-01-15; **Record Level:** language: en; collectionCode: KSMA; datasetName: Ants; ownerInstitutionCode: KSU; basisOfRecord: PreservedSpecimen
- **Type status:** Other material. **Occurrence:** recordedBy: M.R. Sharaf; individualCount: 3; sex: worker; lifeStage: adult; **Taxon:** scientificName: Tapinoma simrothi; **Location:** country: Saudi Arabia; locality: Wadi Hanifa; verbatimElevation: 641 m; decimalLatitude: 24.67088; decimalLongitude: 46.59513; georeferenceProtocol: label; **Identification:** identifiedBy: Mostafa R. Sharaf; dateIdentified: 2013; **Event:** samplingProtocol: Hand picking; eventDate: 2013-04-11; **Record Level:** language: en; collectionCode: KSMA; datasetName: Ants; ownerInstitutionCode: KSU; basisOfRecord: PreservedSpecimen
- **Type status:** Other material. **Occurrence:** recordedBy: S. Salman; individualCount: 1; sex: worker; lifeStage: adult; **Taxon:** scientificName: Tapinoma simrothi; **Location:** country: Saudi Arabia; locality: Wadi Hanifa; verbatimElevation: 654 m; decimalLatitude: 24.67089; decimalLongitude: 46.58061; georeferenceProtocol: label; **Identification:** identifiedBy: Mostafa R. Sharaf; dateIdentified: 2014; **Event:** samplingProtocol: Pitfall trap; eventDate: 2014-02-14; **Record Level:** language: en; collectionCode: KSMA; datasetName: Ants; ownerInstitutionCode: KSU; basisOfRecord: PreservedSpecimen
- **Type status:** Other material. **Occurrence:** recordedBy: S. Salman; individualCount: 1; sex: worker; lifeStage: adult; **Taxon:** scientificName: Tapinoma simrothi; **Location:** country: Saudi Arabia; locality: Wadi Hanifa; verbatimElevation: 684 m; decimalLatitude: 24.74123; decimalLongitude: 46.56113; georeferenceProtocol: label; **Identification:** identifiedBy: Mostafa R. Sharaf; dateIdentified: 2014; **Event:** samplingProtocol: Pitfall trap; eventDate: 2014-09-18; **Record Level:** language: en; collectionCode: KSMA; datasetName: Ants; ownerInstitutionCode: KSU; basisOfRecord: PreservedSpecimen
- **Type status:** Other material. **Occurrence:** recordedBy: S. Salman; individualCount: 3; sex: worker; lifeStage: adult; **Taxon:** scientificName: Tapinoma simrothi; **Location:** country: Saudi Arabia; locality: Wadi Hanifa; verbatimElevation: 674 m; decimalLatitude: 24.73507; decimalLongitude: 46.57518; georeferenceProtocol: label; **Identification:** identifiedBy: Mostafa R. Sharaf; dateIdentified: 2014; **Event:** samplingProtocol: Pitfall trap; eventDate: 2014-09-18; **Record Level:** language: en; collectionCode: KSMA; datasetName: Ants; ownerInstitutionCode: KSU; basisOfRecord: PreservedSpecimen
- **Type status:** Other material. **Occurrence:** recordedBy: S. Salman; individualCount: 4; sex: worker; lifeStage: adult; **Taxon:** scientificName: Tapinoma simrothi; **Location:** country: Saudi Arabia; locality: Wadi Janadriya; verbatimElevation: 603 m; decimalLatitude: 24.8773; decimalLongitude: 46.82031; georeferenceProtocol: label; **Identification:** identifiedBy: Mostafa R. Sharaf; dateIdentified: 2014; **Event:** samplingProtocol: Hand picking; eventDate: 2014-11-19; **Record Level:** language: en; collectionCode: KSMA; datasetName: Ants; ownerInstitutionCode: KSU; basisOfRecord: PreservedSpecimen
- **Type status:** Other material. **Occurrence:** recordedBy: M.R. Sharaf; individualCount: 4; sex: worker; lifeStage: adult; **Taxon:** scientificName: Tapinoma simrothi; **Location:** country: Saudi Arabia; locality: Wadi Qarina; verbatimElevation: 633 m; decimalLatitude: 25.9365; decimalLongitude: 46.833; georeferenceProtocol: label; **Identification:** identifiedBy: Mostafa R. Sharaf; dateIdentified: 2009; **Event:** samplingProtocol: Pitfall trap; eventDate: 2009-11-05; **Record Level:** language: en; collectionCode: KSMA; datasetName: Ants; ownerInstitutionCode: KSU; basisOfRecord: PreservedSpecimen
- **Type status:** Other material. **Occurrence:** recordedBy: S. Salman; individualCount: 4; sex: worker; lifeStage: adult; **Taxon:** scientificName: Tapinoma simrothi; **Location:** country: Saudi Arabia; locality: Zulfi; verbatimElevation: 635 m; decimalLatitude: 26.27192; decimalLongitude: 44.77071; georeferenceProtocol: label; **Identification:** identifiedBy: Mostafa R. Sharaf; dateIdentified: 2014; **Event:** samplingProtocol: Pitfall trap; eventDate: 2014-01-18; **Record Level:** language: en; collectionCode: KSMA; datasetName: Ants; ownerInstitutionCode: KSU; basisOfRecord: PreservedSpecimen
- **Type status:** Other material. **Occurrence:** recordedBy: S. Salman; individualCount: 12; sex: worker; lifeStage: adult; **Taxon:** scientificName: Tapinoma simrothi; **Location:** country: Saudi Arabia; locality: Zulfi; verbatimElevation: 623 m; decimalLatitude: 26.27996; decimalLongitude: 44.80443; georeferenceProtocol: label; **Identification:** identifiedBy: Mostafa R. Sharaf; dateIdentified: 2014; **Event:** samplingProtocol: Pitfall trap; eventDate: 2014-01-18; **Record Level:** language: en; collectionCode: KSMA; datasetName: Ants; ownerInstitutionCode: KSU; basisOfRecord: PreservedSpecimen
- **Type status:** Other material. **Occurrence:** recordedBy: M.R. Sharaf; individualCount: 1; sex: queen; lifeStage: adult; **Taxon:** scientificName: Tapinoma simrothi; **Location:** country: Saudi Arabia; locality: Oyaina; verbatimElevation: 749 m; decimalLatitude: 24.90665; decimalLongitude: 46.389917; georeferenceProtocol: label; **Identification:** identifiedBy: Mostafa R. Sharaf; dateIdentified: 2010; **Event:** samplingProtocol: Hand picking; eventDate: 2010-04-28; **Record Level:** language: en; collectionCode: KSMA; datasetName: Ants; ownerInstitutionCode: KSU; basisOfRecord: PreservedSpecimen
- **Type status:** Other material. **Occurrence:** recordedBy: S. Salman; individualCount: 1; sex: queen; lifeStage: adult; **Taxon:** scientificName: Tapinoma simrothi; **Location:** country: Saudi Arabia; locality: Wadi El Dawaser; verbatimElevation: 686 m; decimalLatitude: 22.7774; decimalLongitude: 44.78624; georeferenceProtocol: label; **Identification:** identifiedBy: Mostafa R. Sharaf; dateIdentified: 2015; **Event:** samplingProtocol: Hand picking; eventDate: 2015-02-22; **Record Level:** language: en; collectionCode: KSMA; datasetName: Ants; ownerInstitutionCode: KSU; basisOfRecord: PreservedSpecimen

#### Diagnosis

**Worker.** Head in full-face view with strongly convex sides and nearly straight posterior margin (Fig. 3c); anterior central margin with a median well-defined notch, deeper than broad; with head in full-face view, scape surpassing posterior margin of head by about one third of its length (Fig. 3c); metanotal groove distinct (Fig. 3a); propodeal dorsum short, meeting declivity at an obtuse angle; colour uniform dark brown to black, tarsi yellow-brown; whole body covered with appressed pubescence (Fig. 3a, b).

##### Previous records

**KUWAIT**: Failaka Island; Kuwait; Wadi Umm al-Rumam (Collingwood & Agosti 1996). **OMAN**: Wahiba Sands (Collingwood 1985). **SA**: Al Qatif (Collingwood 1985). **UAE**: Al-Ain (Collingwood et al. 2011). **YEMEN**: AI-Mahwit (Collingwood and Agosti 1996).

#### Distribution

A Palearctic species (Borowiec 2014) originally described from Italy. It is a broadly distributed species in the Arabian Peninsula (Collingwood 1985, Collingwood and Agosti 1996, Collingwood et al. 2011, Sharaf et al. 2018), Egypt (Sharaf 2006), Iran (Pashaei Rad et al. 2018) and Turkey (Kiran and Karaman 2012).

#### Ecology

In SA, *T. simrothi* was found nesting amongst roots of lawns, attending mealybugs and co-existing with *Solenopsis abdita* (Sharaf and Aldawood 2011). The species also attends *Aphis gossypii* Glover, 1877 for honeydew and the latter gains protection from predators in return (Karami-jamour et al. 2018). The species was nesting under stones next to *Acacia* trees of the desert habitats of the Riyadh Province.

### Tapinoma wilsoni

Sharaf & Aldawood, 2012

1540266A-94D4-5AFE-BCC6-503D3FE13941

https://www.gbif.org/species/8745285

Tapinoma wilsoni Sharaf & Aldawood, 2012c: 38, figs. 1-3 (w.) Saudi Arabia. Afrotropic.
Tapinoma wilsoni Sharaf & Aldawood, 2012c: 38, figs. 1-3 (w.) Saudi Arabia. Afrotropic.

#### Materials

- **Type status:** Other material. **Occurrence:** recordedBy: M.R. Sharaf; individualCount: 1; sex: worker; lifeStage: adult; **Taxon:** scientificName: Tapinoma wilsoni; **Location:** country: Saudi Arabia; locality: Dhi Ayn Archaeological Village; verbatimElevation: 741 m; decimalLatitude: 19.9297; decimalLongitude: 41.4427; georeferenceProtocol: label; **Identification:** identifiedBy: Mostafa R. Sharaf; dateIdentified: 2012; **Event:** samplingProtocol: Hand picking; eventDate: 2011-05-15; **Record Level:** language: en; collectionCode: KSMA; datasetName: Ants; ownerInstitutionCode: KSU; basisOfRecord: PreservedSpecimen
- **Type status:** Other material. **Occurrence:** recordedBy: M.R. Sharaf; individualCount: 29; sex: worker; lifeStage: adult; **Taxon:** scientificName: Tapinoma wilsoni; **Location:** country: Saudi Arabia; locality: Dhi Ayn Archaeological Village; verbatimElevation: 741 m; decimalLatitude: 19.9297; decimalLongitude: 41.4427; georeferenceProtocol: label; **Identification:** identifiedBy: Mostafa R. Sharaf; dateIdentified: 2012; **Event:** samplingProtocol: Hand picking; eventDate: 2011-05-15; **Record Level:** language: en; collectionCode: KSMA; datasetName: Ants; ownerInstitutionCode: KSU; basisOfRecord: PreservedSpecimen
- **Type status:** Other material. **Occurrence:** recordedBy: A. Polaszek; individualCount: 3; sex: worker; lifeStage: adult; **Taxon:** scientificName: Tapinoma wilsoni; **Location:** country: Saudi Arabia; locality: Dhi Ayn Archaeological Village; verbatimElevation: 741 m; decimalLatitude: 19.9297; decimalLongitude: 41.4427; georeferenceProtocol: label; **Identification:** identifiedBy: Mostafa R. Sharaf; dateIdentified: 2015; **Event:** samplingProtocol: Yellow pan trap; eventDate: 2015-04-11; **Record Level:** language: en; collectionCode: KSMA; datasetName: Ants; ownerInstitutionCode: KSU; basisOfRecord: PreservedSpecimen
- **Type status:** Other material. **Occurrence:** recordedBy: F.S. Esteves; individualCount: 1; sex: queen; lifeStage: adult; **Taxon:** scientificName: Tapinoma wilsoni; **Location:** country: Saudi Arabia; locality: Dhi Ayn Archaeological Village; verbatimElevation: 735 m; decimalLatitude: 19.9297; decimalLongitude: 41.4427; georeferenceProtocol: label; **Identification:** identifiedBy: Mostafa R. Sharaf; dateIdentified: 2012; **Event:** samplingProtocol: none specified; eventDate: 2011-09-23; **Record Level:** language: en; collectionCode: CASC; basisOfRecord: PreservedSpecimen
- **Type status:** Other material. **Occurrence:** recordedBy: M.R. Sharaf; individualCount: 4; sex: worker; lifeStage: adult; **Taxon:** scientificName: Tapinoma wilsoni; **Location:** country: Saudi Arabia; locality: Dhi Ayn Archaeological Village; verbatimElevation: 728 m; decimalLatitude: 19.9297; decimalLongitude: 41.4427; georeferenceProtocol: label; **Identification:** identifiedBy: Mostafa R. Sharaf; dateIdentified: 2016; **Event:** samplingProtocol: Hand picking; eventDate: 2016-04-11; **Record Level:** language: en; collectionCode: KSMA; datasetName: Ants; ownerInstitutionCode: KSU; basisOfRecord: PreservedSpecimen

#### Diagnosis

**Worker.** Head in full-face view longer than broad with shallowly convex posterior margin and sides; anterior clypeal margin broadly and distinctly concave; scapes, in full-face view, surpass posterior margin of head by about 1/6 of its length; all funicular segments distinctly longer than broad (Fig. 4c); metanotal groove indistinct; propodeum in profile with the transition from dorsum to declivity sharply defined, the declivity concave and the angle with a raised apex (Fig. 4a); colour yellow to brown-yellow, antennae and legs uniform yellow (Fig. 4a, b).

##### Previous records

**SA**: Al Bahah, Dhi Ayn Archaeological Village, 19.9297°N, 41.4427°E, 741 m alt. (Sharaf et al. 2012)

#### Distribution

Saudi Arabia.

#### Ecology

*Tapinoma wilsoni* was found foraging on the ground surface of a Banana farm in Dhi Ayn Archeological Village, a semi-isolated area that is completely surrounded by high mountains of the Al Sarawat Mountains (KSA) and the soil is clay and humid (Sharaf et al. 2012). Nothing is known about the biology of the species. The species is co-existing with the ant species *Carebara arabica* (Collingwood & van Harten, 2011), *Tetramorium sericeiventre* Emery, 1877, *Pheidole minuscula* Bernard, 1953, *Trichomyrmex destructor* (Jerdon, 1851) and *Monomorium exiguum* Forel, 1894.

## Identification Keys

### Key to the Arabian species of the genus *Tapinoma*

**Table:** 

1	Propodeum in profile with the transition from dorsum to declivity sharply defined, the declivity concave and the angle with a raised apex (Fig. 5a)	*T. wilsoni* Sharaf & Aldawood
–	Propodeum in profile with the transition from dorsum to declivity has a rounded angle (Fig. 5b)	2
2	Larger species, TL more than 2.0 mm; anterior clypeal margin with a deep median notch (Fig. 5c); colour darker, uniformly blackish-brown or black	*T. simrothi* Krausse
–	Smaller species, TL less than 2.0 mm; anterior clypeal margin with only a shallow median concavity (Fig. 5d); head and mesosoma dark yellowish-brown, gaster yellow	*T. melanocephalum* (Fabricius)

## Discussion

**Figure 6.**
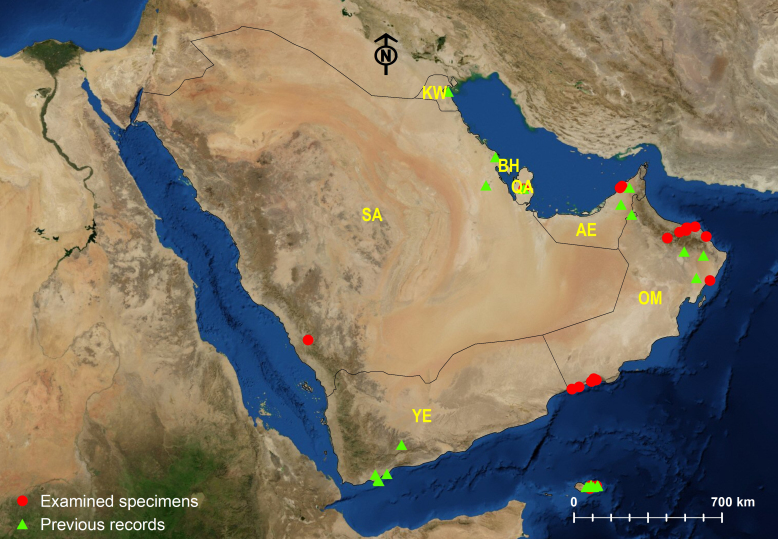
Distribution map of *Tapinoma melanocephalum*.

**Figure 7.**
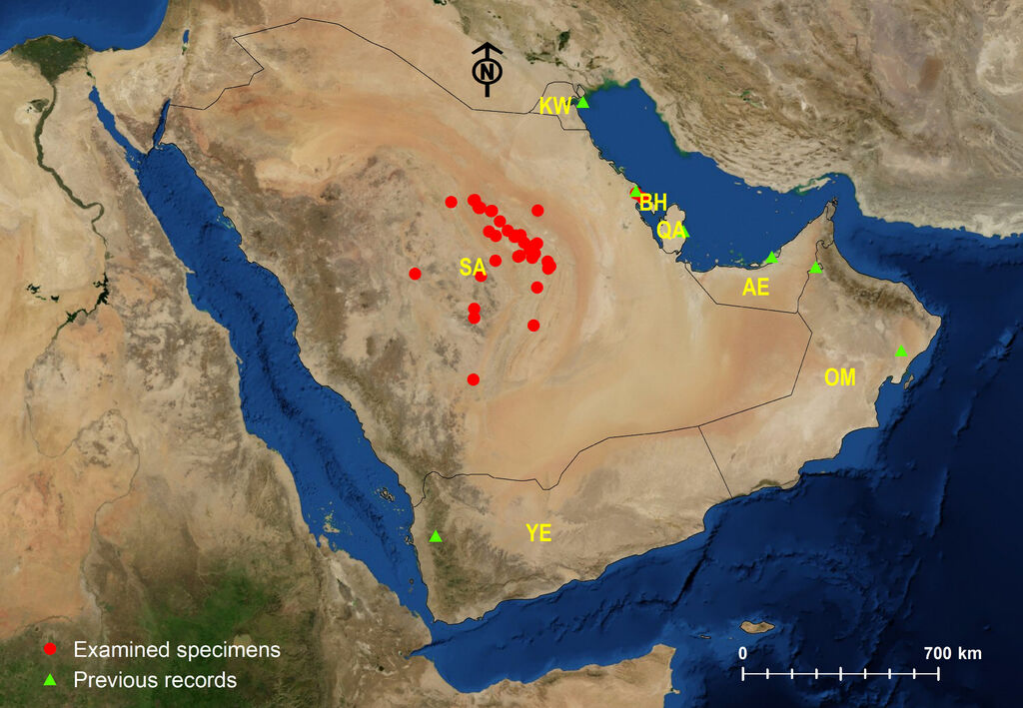
Distribution map of *Tapinoma simrothi*.

**Figure 8.**
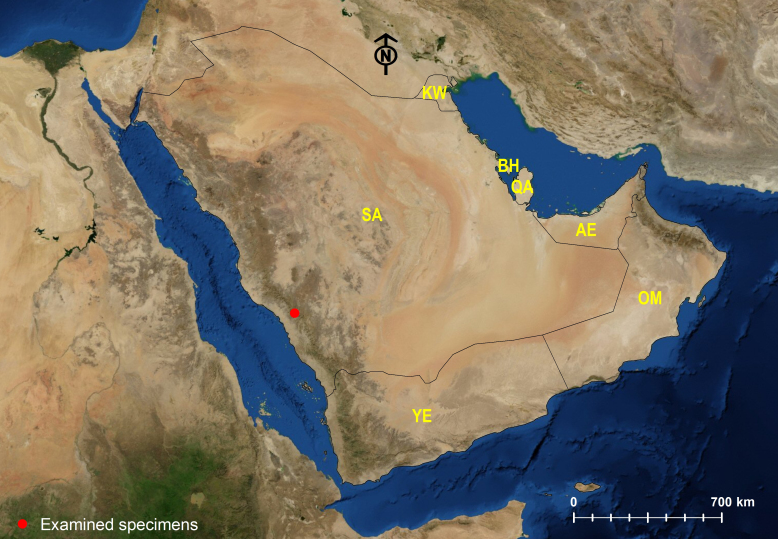
Distribution map of *Tapinoma wilsoni*.

Amongst the three Arabian *Tapinoma* species, *T. simrothi* is the most widespread species, followed by *T. melanocephalum*, whereas *T. wilsoni* is the rarest species with a limited geographic distribution confined to the type locality (Dhi Ayn Archeological Village) at the Al Sarawat Mountains, KSA) (Figs 6, 7, 8). The wide geographic distribution of *T. melanocephalum* is related to the broad habitat preference of the species (Smith 1965, Oster and Wilson 1978, Venkataramaiah and Rehman 1989, Hölldobler and Wilson 1990, Smith and Whitman 1992, Wetterer 2009, Ellison et al. 2012, Sharaf et al. 2017). *Tapinoma simrothi* is broadly distributed, especially in public gardens and agricultural fields of the Central Region of the Arabian Peninsula and with apparent associations with the sap-sucking insects for honeydew (Collingwood et al. 2011, Sharaf and Aldawood 2011). The species is also widely spread in the wild sites of the deserts in Riyadh Province, especially near to *Acacia* trees. The ability of species to inhabit both urban and wild habitats of the Arabian Peninsula identifies the wide geographic distribution.

## Supplementary Material

5B120DBB-8D23-5EB9-B869-A2762860EC14 10.3897/BDJ.9.e66058.suppl1 Supplementary material 1 Appendix 1. Material examined data Data type Occurrences Brief description Distribution data of the examined material File: oo_518847.xlsx https://binary.pensoft.net/file/518847 Mahmoud S. Abdel-Dayem, Hathal M. Aldhafer, Abdulrahman S. Aldawood, Mostafa R. Sharaf

XML Treatment for Tapinoma melanocephalum

XML Treatment for Tapinoma simrothi

XML Treatment for Tapinoma wilsoni

## Figures and Tables

**Figure 2a.**
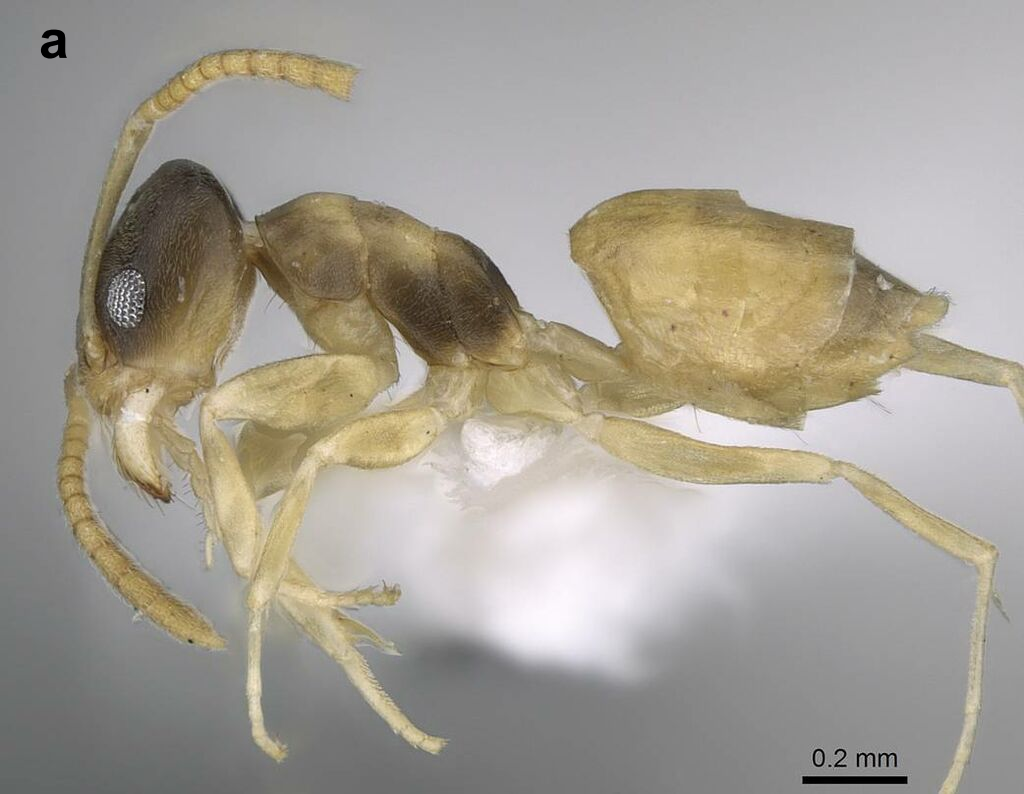
body in profile.

**Figure 2b.**
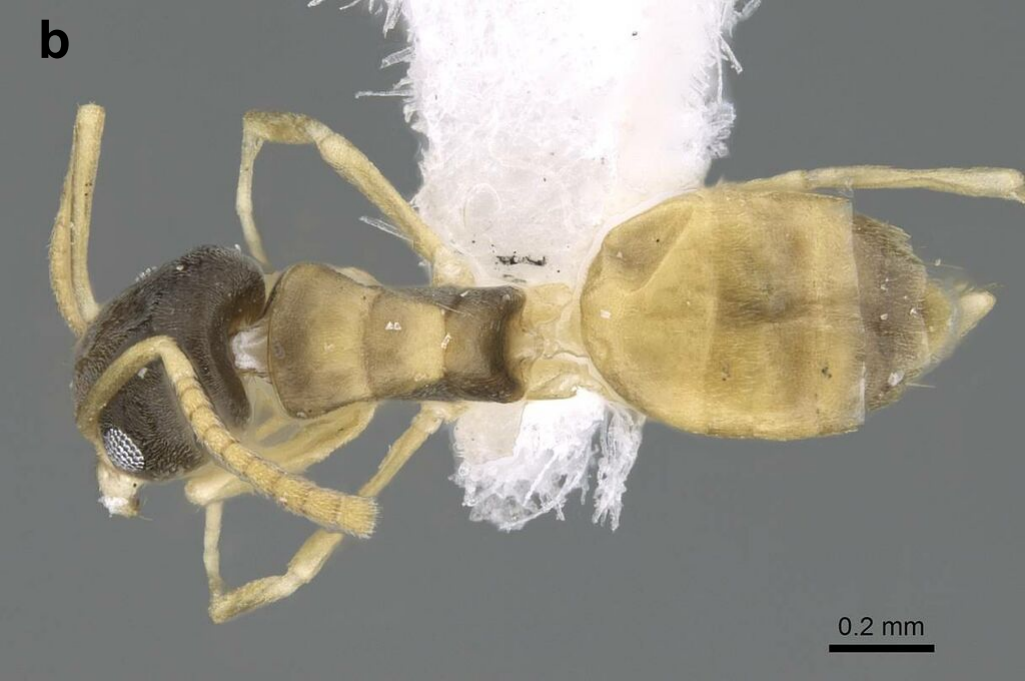
body in dorsal view.

**Figure 2c.**
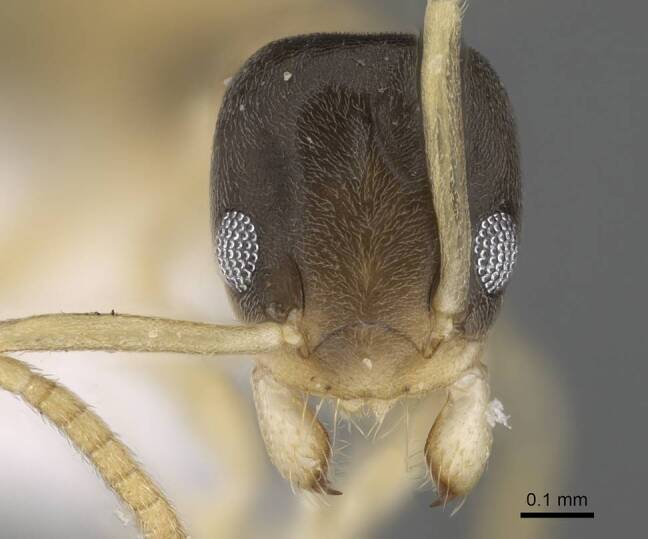
head in full-face view.

**Figure 3a.**
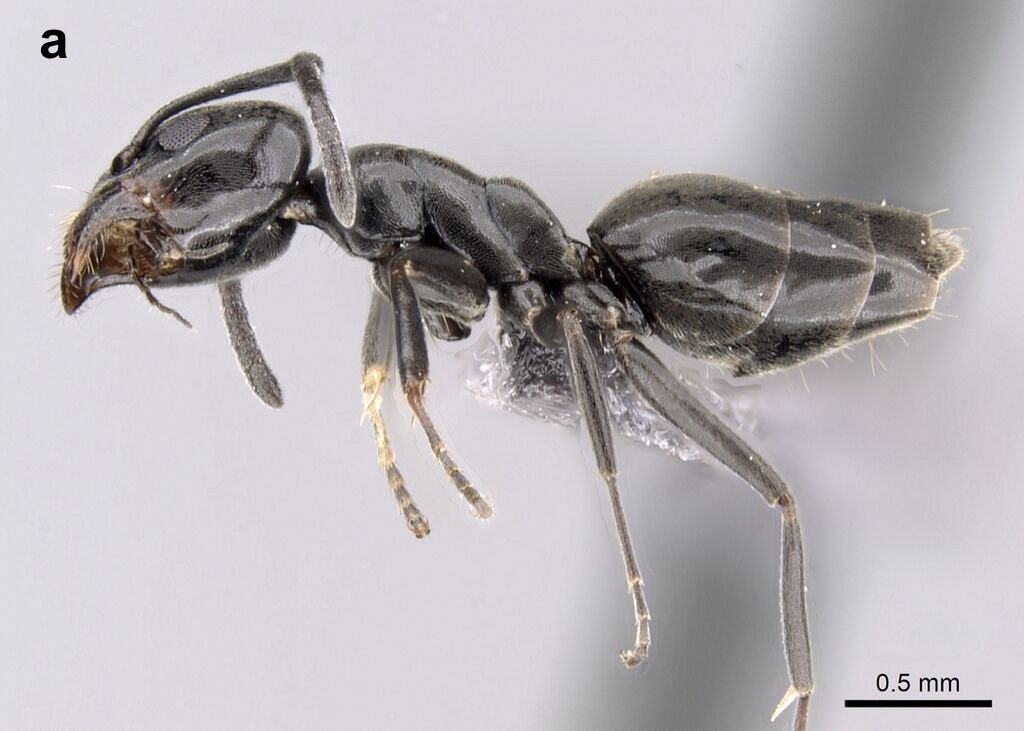
body in profile.

**Figure 3b.**
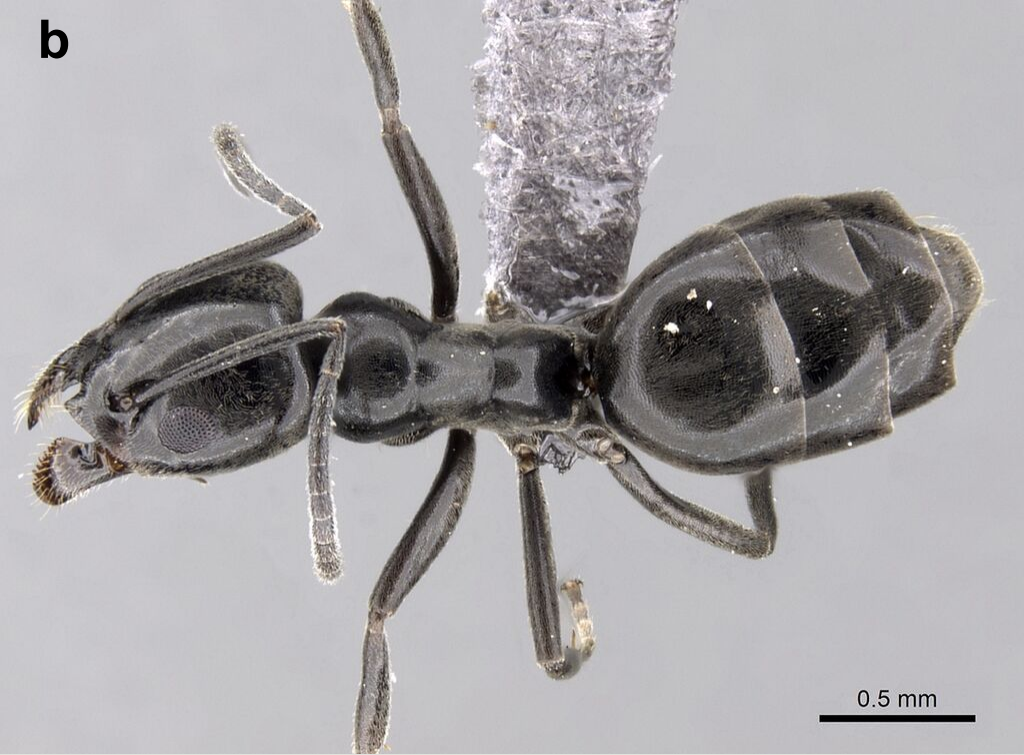
body in dorsal view.

**Figure 3c.**
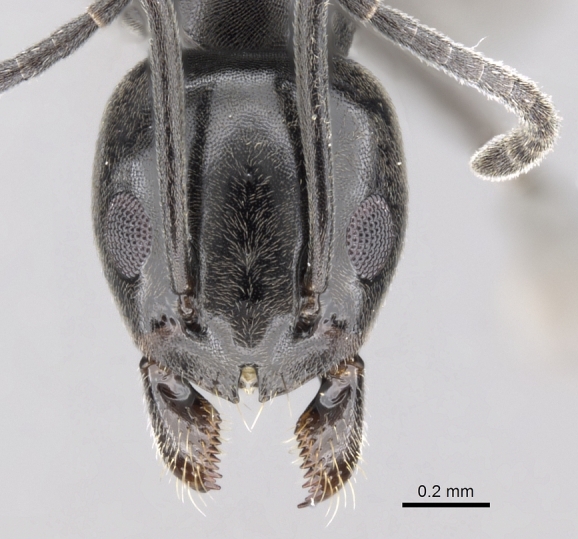
head in full-face view.

**Figure 4a.**
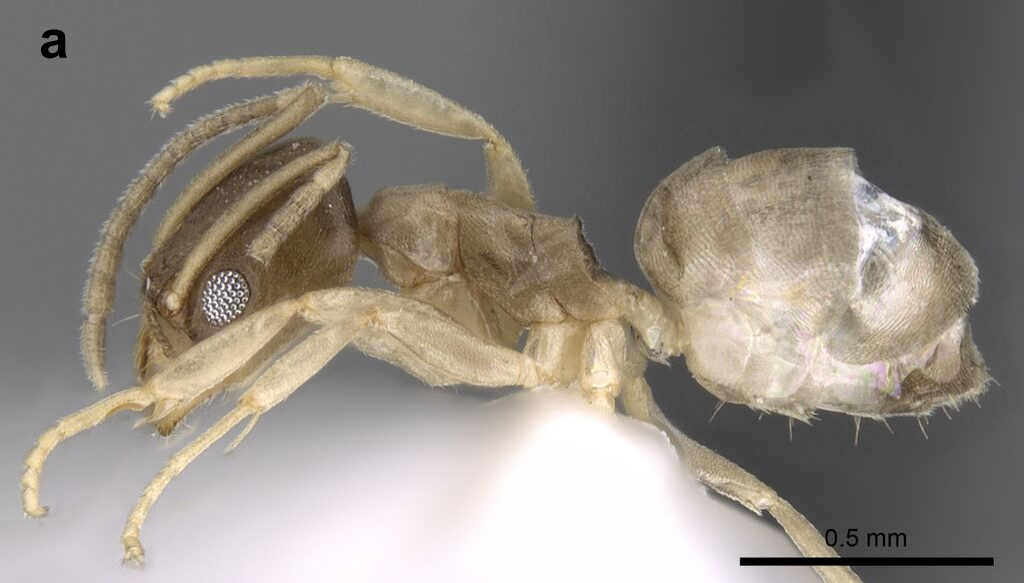
body in profile.

**Figure 4b.**
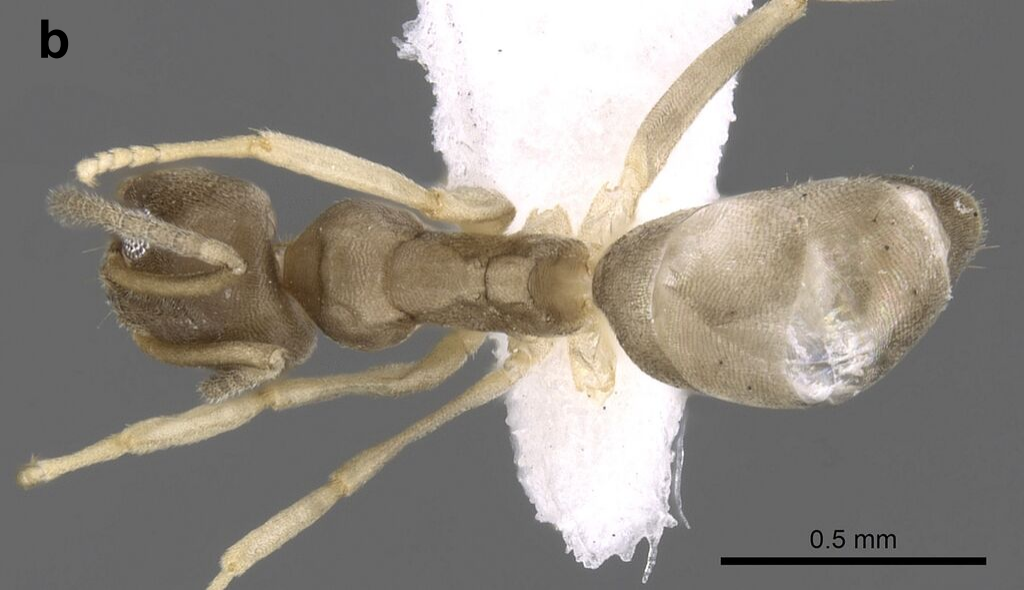
body in dorsal view.

**Figure 4c.**
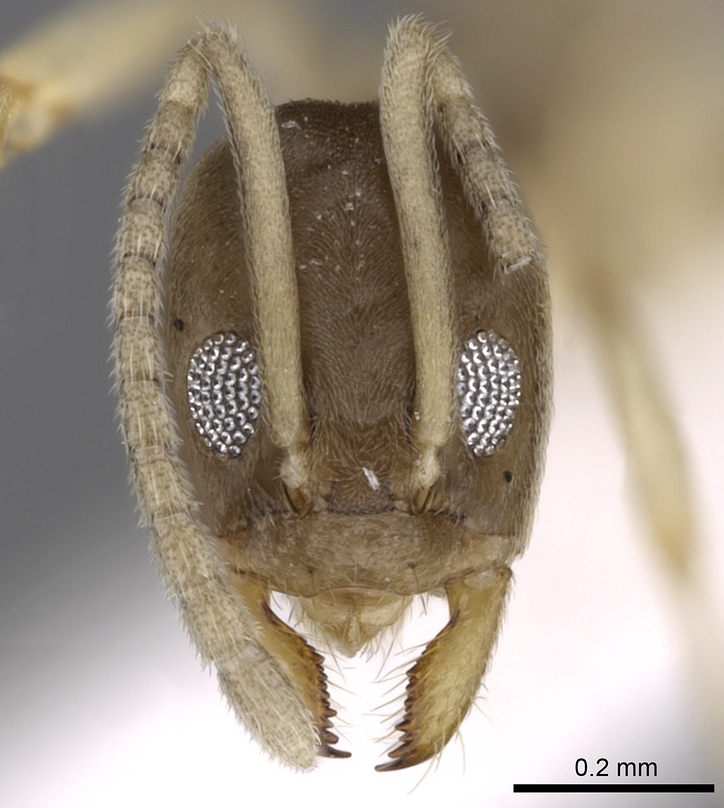
head in full-face view.

**Figure 5a.**
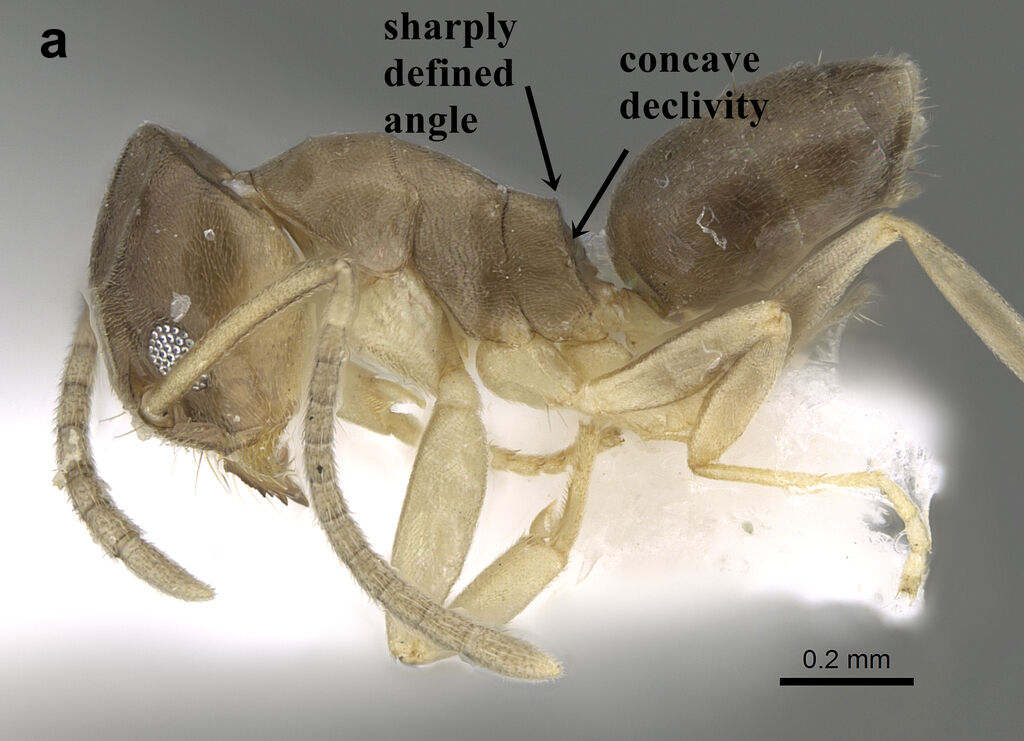
*Tapinoma wilsoni* profile showing the concave declivity and sharply defined raised angle, CASENT0919800.

**Figure 5b.**
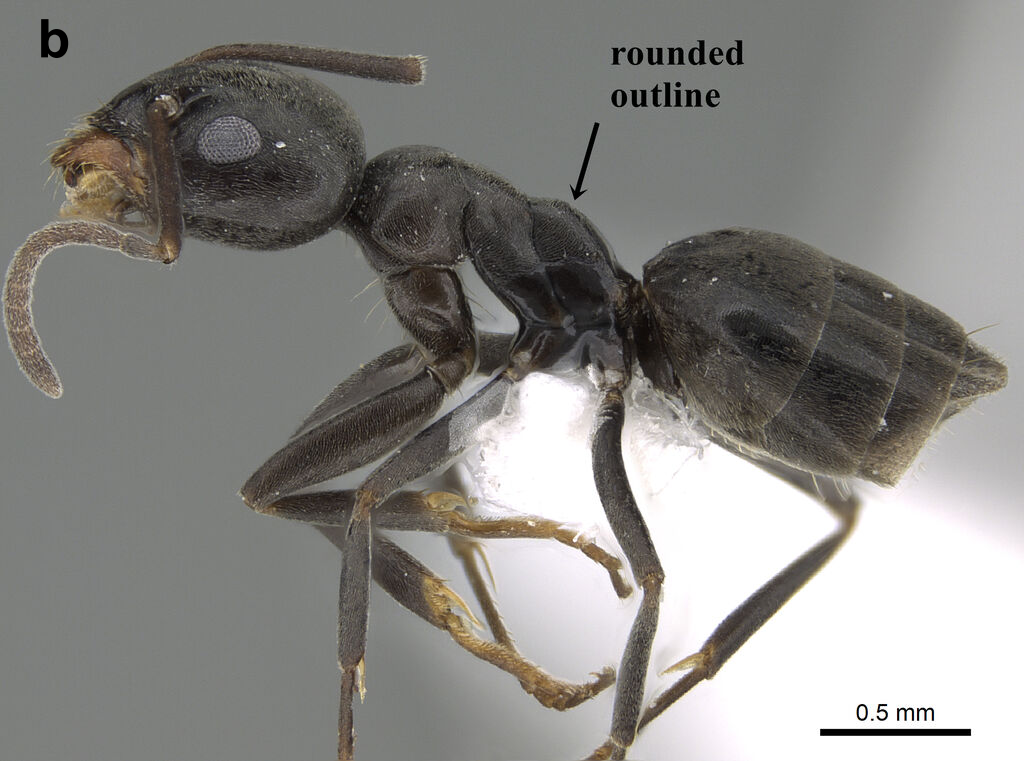
*Tapinoma imrothi* profile showing the rounded outline between propodeal dorsum and declivity, CASENT0919801.

**Figure 5c.**
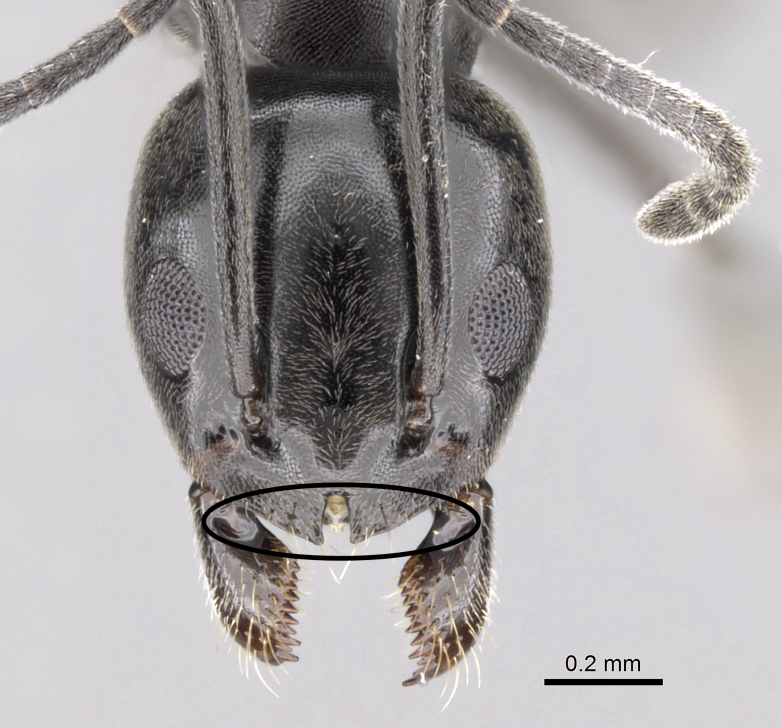
head in full-face view of *Tapinoma simrothi* showing the median clypeal notch, CASENT0919801.

**Figure 5d.**
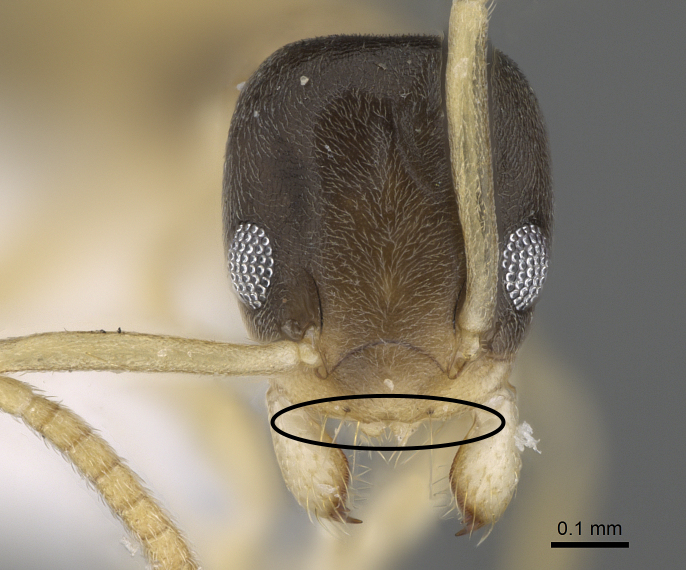
head in full-face view of *Tapinoma melanocephalum* showing the absence of the clypeal notch, CASENT0922277.
